# Genes Contributing to the Unique Biology and Intrinsic Antibiotic Resistance of Enterococcus faecalis

**DOI:** 10.1128/mBio.02962-20

**Published:** 2020-11-24

**Authors:** Michael S. Gilmore, Rauf Salamzade, Elizabeth Selleck, Noelle Bryan, Suelen S. Mello, Abigail L. Manson, Ashlee M. Earl

**Affiliations:** a Department of Ophthalmology, Harvard Medical School and Mass Eye and Ear, Boston, Massachusetts, USA; b Department of Microbiology, Harvard Medical School and Mass Eye and Ear, Boston, Massachusetts, USA; c Infectious Disease and Microbiome Program, Broad Institute of MIT and Harvard, Cambridge, Massachusetts, USA

**Keywords:** *Enterococcus faecalis*, antibiotic resistance, evolutionary biology, genomes, metabolism, cell wall, toxin/antitoxin systems, two-component signal

## Abstract

The enterococci, which are among the leading causes of multidrug-resistant (MDR) hospital infection, are notable for their environmental ruggedness, which extends to intrinsic antibiotic resistance. To identify genes that confer this unique property, we used Tn-seq to comprehensively explore the genome of MDR Enterococcus faecalis strain MMH594 for genes important for growth in nutrient-containing medium and with low-level antibiotic challenge. As expected, a large core of genes for DNA replication, expression, and central metabolism, shared with other bacteria, are intolerant to transposon disruption. However, genes were identified that are important to E. faecalis that are either absent from or unimportant for Staphylococcus aureus and Streptococcus pneumoniae fitness when similarly tested. Further, 217 genes were identified that when challenged by sub-MIC antibiotic levels exhibited reduced tolerance to transposon disruption, including those previously shown to contribute to intrinsic resistance, and others not previously ascribed this role. E. faecalis is one of the few Gram-positive bacteria experimentally shown to possess a functional Entner-Doudoroff pathway for carbon metabolism, a pathway that contributes to stress tolerance in other microbes. Through functional genomics and network analysis we defined the unusual structure of this pathway in E. faecalis and assessed its importance. These approaches also identified toxin-antitoxin and related systems that are unique and active in E. faecalis. Finally, we identified genes that are absent in the closest nonenterococcal relatives, the vagococci, and that contribute importantly to fitness with and without antibiotic selection, advancing an understanding of the unique biology of enterococci.

## Abstract (precis)

Enterococci are leading causes of antibiotic-resistant infection transmitted in hospitals. The intrinsic hardiness of these organisms allows them to survive disinfection practices and then proliferate in the gastrointestinal tracts of antibiotic-treated patients. The objective of this study was to identify the underlying genetic basis for its unusual hardiness. Using a functional genomic approach, we identified traits and pathways of general importance for enterococcal survival and growth that distinguish them from closely related pathogens as well as ancestrally related species. We further identified unique traits that enable them to survive antibiotic challenge, revealing a large set of genes that contribute to intrinsic antibiotic resistance and a smaller set of uniquely important genes that are rare outside enterococci.

## INTRODUCTION

The intrinsic hardiness of enterococci was noted as remarkable over 120 years ago (1). We recently provided evidence that events ∼425 million years ago, likely the terrestrialization of animals, selected for the environmental ruggedness that promoted their survival, transmission, and diversification. The ability of enterococci to survive desiccation and starvation and to be transmitted efficiently in an arid environment positioned them well millions of years later to survive hospital disinfection and antimicrobial regimens (2). Two *Enterococcus* species, Enterococcus faecalis and Enterococcus faecium, now rank among leading causes of multidrug-resistant hospital infection (3, 4).

The molecular basis for this intrinsic hardiness is largely unknown. Enterococci possess novel variations on pathways present in other bacteria, including peptidoglycan biosynthesis (5, 6) and synthesis of other cell wall polymers (7–10), as well as stress response pathways (11), which undoubtedly contribute. It is also likely that genes, and potentially pathways, that are rare outside enterococci and lack correlates in well-studied model organisms (2) also contribute to the intrinsic ruggedness of these bacteria. We previously identified a set of genes that distinguished environmentally resistant enterococci from the closely related vagococci, a group of less resistant microbes often found in marine environments as fish gut commensals (2). To comprehensively delineate which shared and unique traits endow enterococci with their unusual ruggedness, we undertook a functional Tn-seq screen of the E. faecalis genome, combined with a comparative genomics survey of enterococci and close relatives, and identified genes uniquely important to E. faecalis with and without antibiotic challenge.

## RESULTS AND DISCUSSION

### Characteristics and analysis of a *mariner* insertion library in E. faecalis MMH594.

We constructed a high-complexity *mariner* transposon insertion library (2) in E. faecalis MMH594, a multidrug-resistant strain that caused an extended hospital ward bacteremia outbreak that lasted more than 3 years (12, 13). Before exploring the roles and contributions of genes on which E. faecalis depends, we first assessed the library for completeness. To ensure the most robust data set possible and test reproducibility, 10 separate cultures of the *mariner* transposon insertion pool were independently inoculated and grown for 12 generations, on different days, in nutritionally replete Mueller-Hinton (MH) broth. The relative abundance of various transposon insertions in the population was inferred from the number of sequence reads for each transposon junction. Using procedures we employed originally for transposon insertion sequencing (Tn-seq) studies of Staphylococcus aureus (14) and refined by improvements to the analytical pipeline, insertions were mapped to genes in the closed E. faecalis MMH594 genome. A new closed genome sequence for that strain was recently determined from a stock (vial B594) that had been archived without passage since 1985 (13).

This analysis identified *mariner* insertions in 38,366 separate locations and yielded highly reproducible results between cultures (*r *> 0.85; Pearson’s correlation). As expected, some genes were intolerant to transposon insertion, even when organisms were grown in a nutritionally supportive environment. Excluding those genes, this corresponds to a mean distance of 82 bp between transposon insertion sites, with 9 different insertion sites on average in a typical MMH594 gene with an average size of 893 bp. Despite that, we observed several local insertion site anomalies, including overrepresentation of a small set of apparent high-abundance insertions. These represent either (i) hot spots for *mariner* insertion for currently unknown reasons, (ii) overrepresented insertions that may have occurred early and were amplified by outgrowth during the process of library selection, or (iii) intrinsic bias in PCR amplification giving the appearance of overrepresentation. We also observed a high abundance of insertions in multicopy plasmids, which, due to multiple target copies as well as lower G+C content and enrichment in TA sites, provide more opportunities for insertion.

A further source of insertion bias was observed that stems from the nature of replication of the bacterial chromosome. The θ structure of the actively dividing bacterial chromosome provides multiple copies of the replication origin and genes on either side of it, connected through replication forks to a single terminus (15). This creates a merodiploid or even polyploid state for genes occurring nearer the origin, effectively enriching that region of DNA in targets for random transposon insertion. This was previously shown to bias the representation of insertions in the mutant pool, favoring insertions in genes with transiently higher copy numbers near the origin (16). We found such replication bias in the MMH594 insertion library (Fig. S1). This implies an average of slightly more than 2 origins per terminus at the time the *mariner* transposon was induced to transpose.

10.1128/mBio.02962-20.1 FIG S1 Frequency of saturation is influenced by replication bias. Fractional TA occupancy across the E. faecalis MMH594 genome in 10-kb windows is shown. Replication bias is known to affect the count of insertions per site (16), and it also affects the frequency of saturation in our library. The blue line represents a local regression fitting, using the LOESS method, with the grey shading showing the confidence interval at 95%. Download FIG S1, TIF file, 2.8 MB. Copyright © 2020 Gilmore et al. 2020 Gilmore et al. This content is distributed under the terms of the Creative Commons Attribution 4.0 International license.

**FIG 1:**
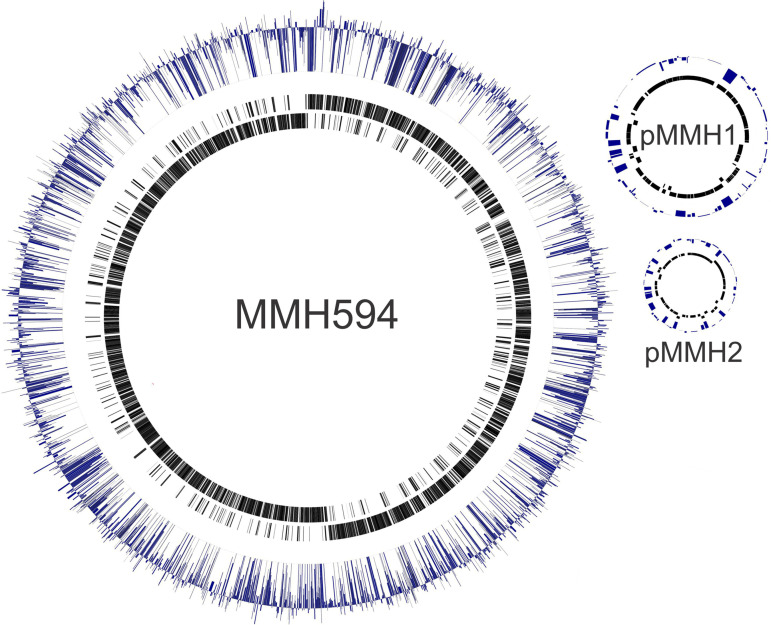
Genes of importance for E. faecalis growth in Mueller-Hinton broth. The 3,152,103-bp chromosome and 2 plasmids (pMMH1 [70.2 kb] and pMMH2 [45.4 kb]) of E. faecalis MMH594. Outer blue lines represent the abundance of transposon junction reads occurring in the central 80% of each gene, normalized to the number of insertion sites within that gene (modified dVal) on a log scale. Blue lines extending above the center line indicate genes possessing insertions represented at a high level in the population (inferring no fitness loss associated with that insertion, or a possible fitness gain), with lines below inferring a potential fitness loss and hence underrepresentation in the population [scale capped: −2 < log_10_(dVal) < 1.24]. Inner black lines show locations of genes on the leading and lagging strands of the MMH594 chromosome and plasmids.

With evidence of multiple insertions in nearly all, if not all, structural genes that will tolerate transposon insertion disruption and still yield a cell capable of growth and division, we next sought to identify those that encode functions that uniquely contribute to E. faecalis fitness, even in the nutritionally supportive environment of Mueller-Hinton broth. To normalize the number of insertions and hence insertion junction reads to gene size, we calculated a variant of the *D* value (dVal) metric previously used (14). Rather than normalizing to gene length (14), we normalized insertions to the number of TA insertion site opportunities within each gene. Furthermore, insertions in the first and last 10% of a reading frame were excluded, so only insertions occurring within the central 80% of each gene were scored. Although it reduced resolution, especially for short genes with few insertion sites and those in areas with low density of insertion, such as near the terminus, this was done to limit noise from insertions that may not be associated with a phenotype. The relative abundance of mutations in MMH594 genes in the mutant population, inferred from dVal, is shown in Fig. 1.

An iterative process was then used to classify genes as highly required for growth (critical), contributory to fitness resulting in some debilitation when mutated (important), or noncontributory in the environment tested (nonessential). In the first step, stringent cutoffs were selected for binning genes based on dVal. Genes in the mutant population represented by 0 or <1% of the expected number of transposon junction reads (dVal < 0.01) were nominated as potentially critical. Genes yielding less than 10% of the expected number of transposon junction reads based on the number of TA insertion opportunities in the middle 80% of the reading frame (dVal < 0.1) were considered potentially important to fitness in the environment tested (i.e., compromised, but to a lesser degree than critical).

**FIG 2:**
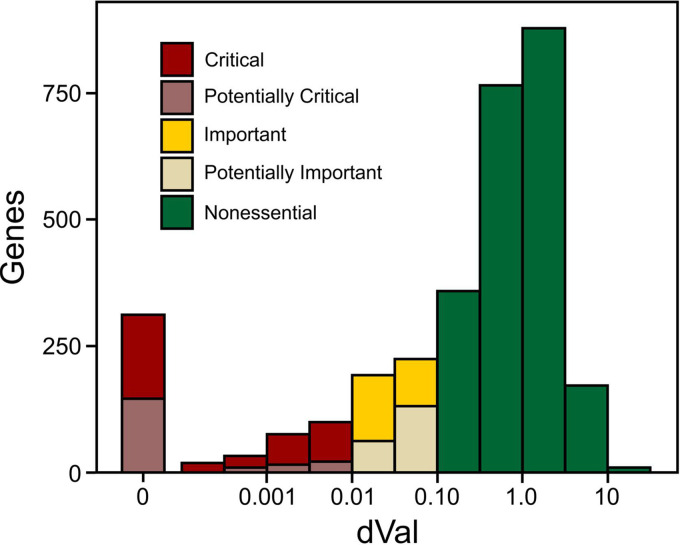
Classification of the effect of transposon insertion on fitness. Of 538 genes with qualifying dVal of <0.01 based on the central 80% of each gene, 349 were validated as Critical by one or more measures (dark red), with 149 remaining unconfirmed and classified as Potentially Critical (lighter red). Similarly, 415 genes exhibited dVal between 0.01 and 0.10, of which 224 were validated by at least one measure as being Fitness Important for growth in Mueller-Hinton broth (dark yellow), with the remaining classified as Potentially Important (light yellow). The remaining 2,173 genes with mid-80% insertions at rates corresponding to a dVal of 0.10 or higher were classified as Nonessential (green).

Promotion from candidacy based on low dVal to classification as Fitness Critical or Fitness Important required additional validation. First, we used a stringent statistical permutation test to assess genes for depletion of transposon insertions relative to the local genomic environment. However, this identified only 36 insertion-depleted genes in the quartile of the chromosome centering on the replication terminus, 33% of the rate for the quartile centering on the origin of replication, with genes in the former being 29% larger on average, suggesting a high false-negative rate from the observed replication bias and possibly other causes. To account for skewing of the local environment from replication bias and essential gene clustering, we performed a second statistical assessment for sets of neighboring genes with low dVals in aggregate. This identified an additional 56 genes in the terminus quartile but still showed limited sensitivity for verifying genes intolerant of disruption, as by using these combined approaches, they occurred at only 45% of the rate found for the origin quartile. To identify other likely false negatives, we then took a third approach and compared our Tn-seq results to studies on two related low-G+C Gram-positive pathogens, Streptococcus pneumoniae (17) and S. aureus (18). Genes with qualifying low dVals (0 to <0.10) for which highly conserved orthologs (defined as reciprocal best [BLAST] hits, RBH, with an E value cutoff of <10e−20) were classified as essential or likely essential in those studies were then considered externally validated. Genes identified by dVal, but for which additional validation was not available, retained the “Potential” designation. By this multilayered orthogonal approach, 349 genes validated by at least one additional criterion were classified as Fitness Critical (of which 317 are protein coding) and 224 genes were similarly classified as Fitness Important (Fig. 2; Data Set S1).

10.1128/mBio.02962-20.2 DATA SET S1 Classification of all MMH594 genes as determined by Tn-seq. MMH594 genes classified by contribution to fitness as determined by Tn-seq. Download Data Set S1, XLSX file, 0.5 MB. Copyright © 2020 Gilmore et al. 2020 Gilmore et al. This content is distributed under the terms of the Creative Commons Attribution 4.0 International license.

### Distinguishing features of enterococci: atypical contributions to fitness.

Of the 349 Fitness Critical genes, 310 are not associated with resident phages or other mobile elements (19, 20) and occur on the core chromosome of MMH594 (Data Set S2a). Of those, 218 have orthologs that are conserved and important for fitness in S. pneumoniae (17) and/or S. aureus (18). Similarly, of the 224 genes categorized as Fitness Important, 124 are in nonvariable core regions of the E. faecalis chromosome and have orthologs that are also important for fitness in one or both of the two nonenterococcal comparator species. As for other species (21), the largest functional group of genes making major contributions to fitness of E. faecalis are genes involved in translation, ribosomal structure, and biogenesis (157 Critical and Important genes; Clusters of Orthologous Groups [COG] category J) (Data Set S2b). Showing that much remains to be learned about low-G+C Gram-positive pathogens, the second largest group of genes of importance for growth, even in a nutritionally replete environment, are genes of unknown function (70 Critical and Important genes; COG category S).

10.1128/mBio.02962-20.3 DATA SET S2 (a) MMH594 gene subset contributing to fitness, (b) classified by COG category, and (c) by unique importance to E. faecalis in contrast to S. aureus and/or S. pneumoniae. Download Data Set S2, XLSX file, 0.1 MB. Copyright © 2020 Gilmore et al. 2020 Gilmore et al. This content is distributed under the terms of the Creative Commons Attribution 4.0 International license.

**FIG 3:**
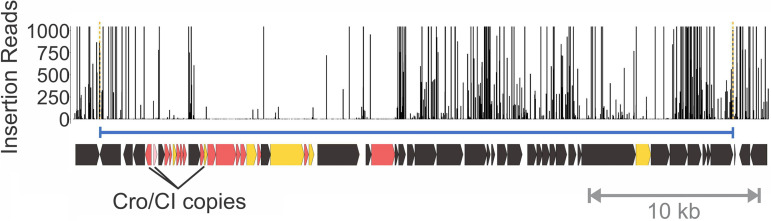
Identification of phage lysogeny control regions as Critical and Important. Vertical lines identify the locations of *mariner* insertions mapped across MMH594/V583 prophage 1, with the height of the line reflecting the number of sequencing reads for that junction in the population. Open reading frames are colored as in Fig. 2: Critical (darker red), Important (darker yellow), Potentially Critical or Important (lighter shades), or Nonessential (black). They are indicated along the length of the phage (blue delimiting line). Genes annotated as Cro/CI copies (EF0306, EF0307, and EF0317) and likely related to lysogeny control and superinfection immunity are indicated. The insertion scale is capped at 1,000.

Genes encoding core functions related to DNA synthesis and chromosomal replication, as well as the translational machinery, are well known to cluster in the bacterial chromosome (22), including in E. faecalis (23). A systematic scan identified 16 MMH594 chromosomal clusters that account for 35.2% of Fitness Critical and 31.3% of Fitness Impairing genes, including the expected core replication, translation, and cell division functions in clusters 1 to 3 (Data Set S3). Unexpectedly, a large group of genes contributing to fitness was identified within prophage *pp1* (20, 24). These included genes annotated as related to lysogeny control (Fig. 3). Derepression of lysogeny is predictably incompatible with bacterial growth, highlighting the diverse ways in which a function can appear in this analysis as conferring fitness. Although categorized as Fitness Critical or Fitness Important, *pp1* and other phage-associated gene clusters were excluded from the list of genes of unique importance to the biology of E. faecalis given that they are not conserved among other E. faecalis lineages (Data Set S3). Despite these phages being enriched for genes of unknown function, their exclusion did not affect the hierarchy of COG functional groups associated with Fitness Critical or Fitness Important genes (Data Set S2b). Chromosomal genes of unknown function remained the second largest COG category.

10.1128/mBio.02962-20.4 DATA SET S3 Clustering of fitness-costly genes of E. faecalis MMH594. Download Data Set S3, XLSX file, 0.01 MB. Copyright © 2020 Gilmore et al. 2020 Gilmore et al. This content is distributed under the terms of the Creative Commons Attribution 4.0 International license.

Identifying phage lysogeny control regions as contributing to fitness in a way that was retrospectively obvious raised the question about other analogous types of fitness contributions, such as that conferred by antitoxins in toxin/antitoxin (TA) modules. Using TADB 2.0 (25), we identified 10 candidate type II TA system pairs in MMH594. One, a hybrid type I/II system consisting of EF3261 to -3263 (Data Set S4), has been validated experimentally (26). TADB 2.0 did not identify the 27-amino-acid-encoding gene EF3263, because it was not annotated in the MMH594 genome, but the entire compound *mazEF/txpA-ratA* system identified in V583 (26) is present without polymorphism in MMH594. For this experimentally characterized MazEF-like system, the rate of transposon insertion in the gene encoding the MazE-type antitoxin was 25.8-fold lower than in the gene for the proposed MazF-like toxin (Data Set S4), showing that it is active in MMH594. Every other TADB 2.0-predicted pair showed a similar, if not greater, bias in the rate of insertion in one partner. Inconsistently, for the EF2534/EF2535 pair, insertion occurred 21-fold more often in the gene identified by the program as encoding the antitoxin, which suggested that in this case, the TADB 2.0 functional prediction is likely reversed. To investigate this further, Phyre2 identified EF2534 as a HEPN-family protein with 99.7% confidence over 85% of its sequence, which is structurally related to so_3166 from the Shewanella oneidensis so_3166/so_3165 toxin/antitoxin pair. Phyre2 found EF2435 to be highly structurally related to bipartite nucleotidyltransferase genes, including so_3165, encoding the catalytic domain (99.9% certainty over 89% length). The structurally similar so_3166/so_3165 toxin/antitoxin system has been studied in molecular detail, including protein structure, function, and assembly (27). That paper also noted the previous misidentification of the nucleotidyltransferase component as the toxin, whereas functional studies invariably demonstrated that the HEPN-family protein was actually the toxin. Structure-function analysis showed that a dimer of the HEPN-family protein possesses RNase activity specific for mRNA and that the actual toxin HEPN-family protein and the antitoxin nucleotidyltransferase form an octomer with that RNase activity blocked (27). This again demonstrated the robustness of the Tn-seq analysis for identifying traits associated with negative selection.

10.1128/mBio.02962-20.5 DATA SET S4 Functional identification of toxin/antitoxin systems in E. faecalis. Download Data Set S4, XLSX file, 1.0 MB. Copyright © 2020 Gilmore et al. 2020 Gilmore et al. This content is distributed under the terms of the Creative Commons Attribution 4.0 International license.

The observed consistency in toxin/antitoxin system analysis highlights the limitation of the stringent cutoffs used for the statistical assignment of importance and the challenge of false negatives—only one antitoxin gene qualified initially for promotion to classification as Critical based on the statistical tests used, although 7 of the 8 non-experimentally explored antitoxin genes possessed dVal insertion rates qualifying them as either Potentially Critical or Potentially Important (with the 8th having a dVal of 0.118, just missing the <0.100 threshold for candidacy). The experimentally tested MazE antitoxin also just missed the cutoff, with a dVal of 0.135. Generally, this limitation stems mainly from the statistical challenge posed by small mean sizes of toxin and antitoxin genes (∼400 and ∼350 bp, respectively), being less than half the mean size of E. faecalis genes and leaving fewer opportunities for transposon insertion into the middle 80% of the gene. However, when mean rates of transposon insertion in toxin and antitoxin genes as reflected by dVal are collectively compared, the difference by *t* test is significant at a level of *P* = 0.001 with the above EF2534/EF2535 assignment reversed, highlighting the importance of taking orthogonal approaches to the analysis of such data sets and the robustness of the data obtained.

Importantly, of the evidently functional (based on insertion bias) 10 TA gene pairs, 8 occur in mobile elements, including 6 in lysogenic phages (Data Set S4). It appears likely that these phage gene pairs play roles in superinfection immunity, as has been previously speculated (28) based on functionally analyzed homologs in closely related lactococci (29). That left EF0379/EF0380 as the only other TA module identified by TADB 2.0 not associated with a known mobile element. That pair exhibits a 42:1 putative toxin/antitoxin insertion bias (Data Set S4), indicating that it is indeed functional. By BLASTP alignment, EF0379 exhibits a high degree of inferred amino acid identity to genes annotated as encoding type II TA death-on-curing proteins in other enterococcal, streptococcal, and related species. EF0379 occurs in about 64% of E. faecalis strains, indicating that it is common but not core to the species. Interestingly, all E. faecium strains examined possess EF0379/EF0380 homologs, as do 42% of other enterococcal species, so it appears widespread among the enterococci. In summary, Tn-seq identified a second category of atypical fitness-conferring genes that include antitoxin components of TA systems, and the EF0379/EF0380 pair represents a second functional TA system in the chromosome of E. faecalis.

Finally, the set of genes uniquely of importance to E. faecalis was further refined by removal of genes with conserved orthologs of importance in the two other closely related pathogens. This resulted in a set of 161 genes uniquely important to E. faecalis (Data Set S2c). This refinement altered the hierarchy of functional groups, now with 37 genes of unknown function (COG S) forming the largest functional group of genes, followed by genes associated with cell wall/membrane/envelope biogenesis (COG M) and carbohydrate transport and metabolism (COG G), traits known to distinguish enterococci from other bacteria (2, 5–10).

Interestingly, gene groups for inorganic ion transport and metabolism (COG P) and coenzyme transport and metabolism (COG H) were also enriched among top functional groups of special importance to E. faecalis (Data Set S2c). From the annotations, many genes of selective importance to E. faecalis relate to iron and molybdenum uptake and incorporation of these metals into proteins. This suggests that enterococci have additional needs and pathways for maintaining intracellular redox balance, compared to S. aureus and S. pneumoniae. E. faecalis lacks most of the electron transport chain (30), depriving it of an important tool for maintaining redox balance within the cell. It possesses genes for a cytochrome BD but lacks the ability to make the necessary heme, so that the apocytochrome is nonfunctional in environments and media lacking hematin. We previously showed that reduced quinols extending through the cell membrane of E. faecalis dispose of excess electrons by making singlet reductions of molecular oxygen, generating 15 to 20 nmol extracellular superoxide anion/10^9^ CFU/min (31), potentially a source of considerable oxidative stress.

### Stress response and related unique attributes of enterococci.

Because of the enrichment in redox-active molybdenum and iron metabolism proteins, we compiled a list of all E. faecalis MMH594 genes that encode (i) known redox activities (usually mediated by metalloenzymes with coordinated iron, molybdenum, and selenium centers), (ii) proteins involved in uptake and metabolism of these metals, or (iii) proteins known to be active in mitigating oxidative stress (11). This identified a set of 185 candidate redox-related genes (Data Set S5). Of those, Tn-seq provided evidence that 59 are important or even critical to fitness for growth in nutrient broth (Table 1), with many organized into expression units for iron, molybdenum, and selenium uptake and incorporation into redox enzymes. Interestingly, of 3 oxidoreductases that incorporate both molybdenum and selenium into active centers (EF1390, EF2563, and EF2570), only EF2563 yielded Tn-seq evidence of importance as tested. EF2563 and genes in the operon associated with it (e.g., EF2563 to -2567 and EF2569 to -2572) occur rarely outside E. faecalis and are not conserved in most other enterococci (Data Set S5; Table 1). In fact, it was previously noted that genes of this group were found only in Enterococcus faecalis and the archaeon Haloarcula marismortui, out of 500 prokaryotic species then queried (32). Collectively, this suggests that the EF2563 system is particularly important for the ability of E. faecalis to manage intracellular redox balance and its unique biology.

**TABLE 1:** Iron, molybdenum, and selenium import and metabolism proteins important for E. faecalis growth in rich medium

B594 gene ID	V583 gene ID	Fitness call^*a*^	Inclusion basis^*b*^	Gene	Annotation	dVal	*Enterococcus* conservation (*n* = 24)	COG functional family
SAW_00098	EF0057	Important AC	UE		ABC transporter permease	0.029	100	P
SAW_00222	EF0237	Critical AC	UE	*ecfA1*	Energy-coupling factor transporter ATP-binding protein EcfA1	0.003	100	R; P
SAW_00224	EF0239	Critical AC	UE		Cobalt transporter	0.002	100	H
SAW_00276	EF0282	Critical ABC	Redox	*fabI*	Enoyl-[acyl-carrier-protein] reductase [NADH] FabI	0.002	100	I
SAW_00341	EF0347	Potentially Important C	Redox UE		Peptide methionine sulfoxide reductase	0.054	8	O
SAW_00446	EF0463	Critical ABC	Ox	*sodA*	Superoxide dismutase [Fe]	0.000	100	P
SAW_00453	EF0470	Important ABC	Redox		Ribonucleotide-diphosphate reductase subunit beta	0.017	100	F
SAW_00454	EF0471	Important ABC	Redox		Ribonucleoside-diphosphate reductase, 2C alpha subunit	0.019	100	F
SAW_00455	EF0472	Important ABC	Fe		NrdI protein	0.061	100	F
SAW_00456	EF0473	Critical AC	Redox	*nrdH*	Ribonucleoside-diphosphate reductase 2,2C NrdH-redoxin	0.000	100	O
SAW_00457	EF0475	Important AC	Fe	*feoA*	Ferrous iron transporter A	0.055	88	P
SAW_00458	EF0476	Important AC	Fe	*feoB*	Ferrous iron transporter B	0.086	88	P
SAW_00817	EF0838	Potentially Critical C	Se UE		l-Seryl-tRNA(Ser) selenium transferase	0.000	75	J
SAW_00902	EF0930	Important ABC	Se		Methionine-tRNA ligase	0.015	100	J
SAW_00975	EF1005	Important ABC	Fe		Iron-dependent repressor	0.014	100	K
SAW_01087	EF1108	Potentially Critical C	Redox UE		Oxidoreductase	0.000	33	C
SAW_01313	EF1338	Critical BC	Ox Trx Se	*trx1*	Thioredoxin-disulfide reductase	0.000	100	O
SAW_01337	EF1364	Critical ABC	Redox		Hydroxymethylglutaryl-CoA reductase 2C degradative	0.001	100	I
SAW_01359	EF1386	Critical AC	Mo UE		formate/nitrite transporter	0.000	92	P
SAW_01361	EF1388	Potentially Important C	Mo UE		NADH-quinone oxidoreductase subunit E	0.028	38	C
SAW_01365	EF1392	Critical AC	Mo UE		Molybdenum cofactor biosynthesis protein C	0.000	17	H
SAW_01366	EF1393	Important AC	Mo UE		Molybdenum cofactor biosynthesis protein A	0.011	17	H
SAW_01367	EF1394	Important AC	Mo		MOSC domain-containing protein	0.015	17	S
SAW_01368	EF1395	Important AC	Mo		Molybdenum cofactor biosynthesis family protein	0.028	17	H
SAW_01377	EF1405	Critical BC	Ox Trx	*trx2*	Thioredoxin	0.000	100	O
SAW_01486	EF1519	Critical AC	UE		Cation transporter E1-E2 family ATPase	0.002	100	P
SAW_01520	EF1557	Critical AC	Redox		4-Hydroxy-tetrahydrodipicolinate reductase	0.000	100	E
SAW_01548	EF1585	Potentially Critical C	Ox UE	*perR*	FUR family transcriptional regulator	0.000	100	P
SAW_01596	EF1639	Potentially Important C	Fe UE		Iron compound ABC transporter ATP-binding protein	0.059	100	H; P
SAW_01638	EF1681	Potentially Critical C	Redox UE		Peptide methionine sulfoxide reductase MsrA	0.001	100	O
SAW_01679	EF1724	Important AC	UE		CBS domain-containing protein	0.020	100	P
SAW_01706	EF1754	Important AC	UE		Phosphate transport system regulatory protein PhoU	0.061	100	P
SAW_01724	EF1773	Potentially Critical C	Redox UE		3-Ketoacyl-(acyl-carrier-protein) reductase	0.000	100	R; Q; I
SAW_01804	EF1859	Critical AC	UE		Pantothenate synthetase	0.003	13	H
SAW_02118	EF2191	Critical AC	Redox		dTDP-4-dehydrorhamnose reductase	0.003	92	M
SAW_02226	EF2357	Potentially Important C	Redox UE		Oxidoreductase	0.017	17	S
SAW_02252	EF2390	Important ABC	Fe		FeS cluster assembly protein SufB	0.029	100	O
SAW_02253	EF2391	Critical BC	Fe		NifU family SUF system FeS assembly protein	0.000	100	O
SAW_02255	EF2393	Important ABC	Fe		FeS assembly protein SufD	0.022	100	O
SAW_02256	EF2394	Important ABC	Fe		FeS assembly ATPase SufC	0.011	100	O
SAW_02382	EF2562	Potentially Important C	Mo UE		Flavodoxin	0.048	100	C
SAW_02383	EF2563	Potentially Critical C	Mo Se UE		YqeB family selenium-dependent molybdenum hydroxylase system protein	0.000	17	O
SAW_02391	EF2571	Critical AC	Mo		Xanthine dehydrogenase accessory factor	0.007	17	O
SAW_02392	EF2572	Potentially Important C	Mo UE		Molybdenum transporter	0.032	17	P
SAW_02394	EF2574	Potentially Critical C	Mo UE		Endoribonuclease L-PSP	0.009	92	V
SAW_02395	EF2575	Potentially Important C	Mo UE		Carbamate kinase	0.020	79	E
SAW_02397	EF2578	Potentially Important C	Mo UE		Peptidase	0.063	54	E
SAW_02400	EF2581	Important AC	Mo Se		Selenate reductase subunit YgfK	0.047	75	R; E
SAW_02526	EF2722	Potentially Important C	Fe UE		l-Serine dehydratase, 2C iron-sulfur-dependent, 2C alpha subunit	0.052	100	E
SAW_02536	EF2733	Important ABC	Redox		UDP-*N*-acetylenolpyruvoylglucosamine reductase	0.011	100	M
SAW_02674	EF2881	Critical ABC	Redox		3-Oxoacyl-[acyl-carrier-protein] reductase	0.000	100	I
SAW_02691	EF2899	Critical AC	Fe		Ferredoxin-NADP reductase	0.000	100	O
SAW_02724	EF2934	Important AC	Mo UE		tRNA sulfurtransferase ThiI	0.051	100	J; H
SAW_02832	EF2976	Potentially Critical C	Se UE		l-Seryl-tRNA(Ser) selenium transferase	0.000	75	J
SAW_02921	EF3072	Critical AC	UE		BioY family protein	0.000	88	H
SAW_02933	EF3084	Important AC	Fe UE		Iron compound ABC transporter permease	0.015	96	P
SAW_02934	EF3085	Important AC	Fe UE		Iron compound ABC transporter permease	0.034	100	P
SAW_03004	EF3164	Important AC	Ox	*msrB*	Peptide methionine sulfoxide reductase MsrB	0.044	96	O
SAW_02264	NA	Important ABC	Redox		Oxa1p cytochrome oxidase export family protein, 3B preprotein translocase subunit YidC	0.063	100	M

a Fitness call: A, validated by permutation test; B, validated by orthology; C, qualifying dVal.

b UE, unique enterococcus, no fitness important ortholog in S. pneumoniae (17) or S. aureus (18); Redox, annotated as involved in redox pathway or reaction; Ox, known to be involved in oxidative stress (95); Fe, annotated as involved in iron uptake or containing coordinated iron; Se, annotated as involved in selenium uptake or containing coordinated selenium; Mo, annotated as involved in molybdenum uptake or containing coordinated molybdenum.

10.1128/mBio.02962-20.6 DATA SET S5 Comprehensive identification and assessment of MMH594 genes related to iron, molybdenum and selenium import and metabolism, and intracellular redox balance. Download Data Set S5, XLSX file, 0.2 MB. Copyright © 2020 Gilmore et al. 2020 Gilmore et al. This content is distributed under the terms of the Creative Commons Attribution 4.0 International license.

More is known about iron uptake and utilization by E. faecalis. A Fur homolog (EF1525) regulates 4 iron uptake systems, EF0188, EF0191–EF0193, EF0475/EF0476, and EF3082–EF3085 (33). Of those, Tn-seq evidence indicates that only EF0475/EF0476 and EF3084/EF3085 contribute importantly to fitness in Mueller-Hinton medium (Data Set S5; Table 1). Of 19 genes associated with oxidative stress (34) (Data Set S5), *sodA* (EF0463), genes for thioredoxins 1 and 2 (EF1338 and EF1405), and potentially also *perR* (EF1585) make critical contributions to fitness, and *msrB* (EF3164) makes an important contribution to fitness (Table 1).

A long-overlooked feature of E. faecalis related to oxidative stress stems from an unusual aspect of its central carbon metabolism. In the late 1950s, it was observed by Sokatch and Gunsalus and by Goddard and Sokatch (35, 36) that E. faecalis was rare in being a Gram-positive bacterium with a functional Entner-Doudoroff (ED) shunt off the pentose phosphate pathway (37). For most marine microbes, the ED pathway is the preferred route for carbon metabolism and in many cases is the sole pathway for metabolizing glucose (38). Moreover, this pathway is essential for coping with oxidative stress (38). It was therefore of interest to determine the extent to which E. faecalis depends on this pathway for carbon metabolism and ultimately whether it contributes to its unusual hardiness.

The canonical ED pathway consists of two core genes encoding the Entner-Doudoroff aldolase (*eda*) and dehydratase (*edd*). The only experimental follow-up to the description of the ED pathway in E. faecalis 50 years ago was an attempt to develop a PCR scheme to uniquely identify enterococci, which identified 2 different genes annotated in the E. faecalis genome with possible roles as the Entner-Doudoroff aldolase (*eda*) (39). In a more recent review of closely related streptococcal metabolism, it was noted that all sequenced streptococcal genomes also harbor genes annotated as ED related but invariably lack the Entner-Doudoroff dehydratase (6-phosphogluconate dehydratase [*edd*]) gene. As a result, it was concluded that a functional ED pathway is unlikely in those bacteria (40).

With biochemical evidence proving that the ED pathway is in fact functional in E. faecalis (35, 36), we first attempted to identify the missing *edd* gene. We used the prototype *edd* gene and protein sequences from Gram-negative Escherichia coli and Zymomonas mobilis, where the pathway is well characterized (37). A search of the NCBI database returned nothing with identity in any E. faecalis genome. A functional ED pathway is also known in the catabolically diverse but high-G+C Gram-positive soil actinomycete Rhodococcus jostii, where nutrient starvation stress leads to its induction by about 50-fold (41). In *R. jostii*, the ED genes occur in an operon that includes RHA1_ro02362 (a putative gluconokinase) and colinear genes RHA1_ro02367 to -69 (respectively, *eda*, *edd*, and *zwf*, encoding a glucose-6-phosphate dehydrogenase that is part of the SoxR oxidative stress regulon of E. coli [42]). Assuming a greater degree of conservation with this Gram-positive organism despite its high G+C content, we probed the genomes of MMH594 and prototype strain V583 for ED pathway orthologs. This identified a conserved ortholog of *zwf*, EF1004 (40.73% amino acid identity over 96% of the inferred length of the protein compared to that encoded by RHA1_ro02369), validating the logic. Interestingly, it also identified three somewhat less conserved homologs of *eda* (RHA1_ro02367), EF0423, EF2266, and EF3134, all exhibiting about 32% amino acid identity over approximately 90% of the protein length. From their multiplicity, this suggests that some aspect of this pathway is important to the physiology of E. faecalis under some condition that occurs in nature. However, despite success identifying multiple *eda* homologs, no identifiable E. faecalis homolog to *edd* (RHA1_ro02368) (or to the putative gluconokinase encoded by RHA1_02362) was found.

**FIG 4:**
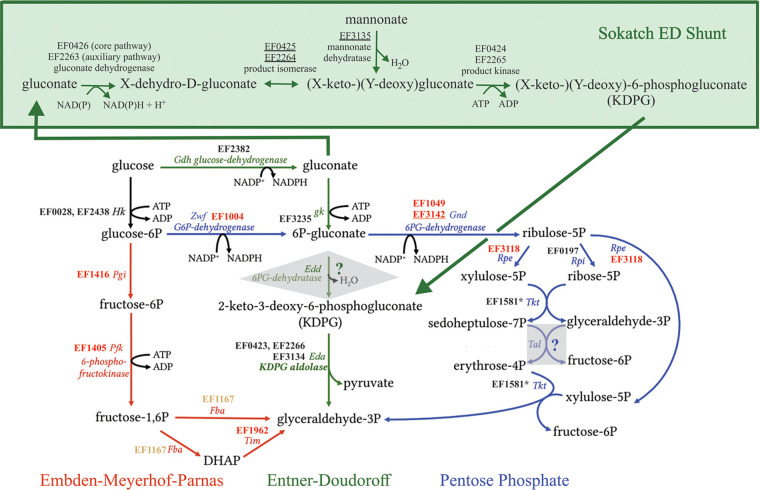
Importance of E. faecalis genes involved in central carbon metabolism. Gene numbers using the V583 convention, corresponding enzymes, and pathways are indicated for carbon metabolism via the Embden-Meyerhof-Parnas pathway (red), the Entner-Doudoroff pathway (green), and the pentose phosphate pathway (violet). Gene EF designations are colored as in Fig. 2 to reflect importance when grown in Mueller-Hinton broth, with red indicating critical for fitness, yellow indicating important for fitness, and black indicating no measurable contribution to fitness as tested. Although long known to possess a functional Entner-Doudoroff pathway (32, 33), E. faecalis lacks the canonical Entner-Doudoroff dehydratase (Edd). Bioinformatic analysis indicates that metabolism is accomplished by a shunt (green box) that distinguishes the E. faecalis activity from that better known for Gram-negative organisms (36).

Collectively, this indicates that E. faecalis must have an alternative to the Edd enzyme for processing gluconate or 6-phosphogluconate to a 2-keto-3-deoxy-6-phosphogluconate-like substrate for one or more of the three Eda homologs (EF0423, EF2266, and EF3134) that occur in MMH594 (Fig. 4). We therefore took a bioinformatic approach to fill this knowledge gap using the three identified *eda* genes as bait. Protein-protein interaction network analysis using STRING (43), through multiple lines of evidence, connected *eda1* EF0423 to the adjacent gene EF0424, which is annotated as a 2-dehydro-3-deoxygluconokinase. This indicates that EF0242 generates the Eda substrate from a nonphosphorylated precursor—an important deviation from the canonical ED pathway, potentially explaining the need for an alternative to Edd (Fig. 4). The same network analysis also connected EF0423 to EF2265 and putative *eda2* EF2266 through multiple lines of evidence. EF2265 is annotated more generically as a carbohydrate kinase, analogous to EF0424, again supporting the prospect that additional phosphorylation is required. Network analysis also showed that EF0423/EF0424 and EF2266/EF2265 both connected to central carbon metabolism through genes associated with Embden-Meyerhof-Parnas (EMP) glycolysis (Fig. 4), as would be expected for an ED-like shunt. EF0424 was found by STRING to connect to three operons: EF0422–EF0426, EF2263–EF2266, and EF3134–EF3135. The EF0422–EF0426 operon encodes a putative transcriptional regulator (EF0422), followed by enzymes that would connect gluconate through to the substrate of the *eda* homolog, EF0423, in reverse ED pathway order, ultimately catalyzing conversion of gluconate to glyceraldehyde-3 phosphate and pyruvate (Fig. 4). The EF2263–EF2266 operon shows an identical organization and is actually a divergent but syntenic copy of the EF0422–EF0426 operon, encoding similar enzymes that range from 65% to 87% in amino acid sequence identity but are conveyed by a vestige of a mobile element that represents a variable region in the MMH594 and V583 chromosomes. This additional operon occurs in select strains of E. faecalis, but outside of that is genetically equidistant from the chromosomal or other enterococcal homologs and that occurring in *Lactobacillus*, suggesting that it likely originates from outside the known enterococci.

The third STRING group connected to the ED pathway is encoded by EF3134/EF3135. As noted, EF3134 corresponds to the third *eda* homolog, *eda3*. However, EF3135 is predicted to encode a mannonate dehydratase, which would generate a keto-deoxy-mannonate product. Whether or how that product becomes phosphorylated to serve as a substrate for EF3134 aldolase cleavage is unknown. It likely either serves as a substrate for phosphorylation by kinases encoded by EF0424/EF2265 or enters the cell by the adjacent phosphotransferase system encoded by EF3136–EF3139 and as a result is already phosphorylated prior to removal of the water molecule by the EF3135 dehydratase. Finally, there are two more divergent *eda*-like enzymes encoded within the E. faecalis genome, EF0839 and EF2975, which are closely related to each other but show no detectable sequence identity to the three mentioned above. EF2975 occurs in the context of genes putatively encoding proteins involved in nitrogen metabolism and nitrogen stress. These pathways may well be involved in the import and processing of amino or other sugars to glyceraldehyde-3-phosphate and pyruvate (or other split products) by an *eda*-type mechanism. All of these findings are incorporated into a new model for a variation of the ED pathway which we term the Sokatch ED shunt after its biochemical discoverer (35) (Fig. 4).

Although the functional ED pathway shown to exist by Sokatch and Gunsalus over 50 years ago (35) includes novel genes, all were found to be dispensable for growth in nutrient broth. One interpretation is that these genes may be dispensable because of their redundancy. E. faecalis strain OG1RF lacks the redundant EF2263–EF2266 pathway, but nevertheless, transposon insertion mutations have been identified in all enzyme-encoding reading frames (44), supporting the prospect that it is in fact dispensable for growth in nutrient broth. Interestingly, Tn-seq provides evidence that EF0839 and EF2975, which may be associated with amino sugar metabolism, may contribute to fitness in nutrient medium.

Although it has not been biochemically explored in the phylogenetically related streptococci, they in fact likely also possess this alternate means for utilizing a modified ED pathway despite the earlier noted lack of an *edd* gene (40). S. aureus lacks homologs of genes encoding enzymes in this shunt, whereas S. pneumoniae contains readily identified orthologs to all of them, except for the isomerases EF0424 and EF2265. Instead, S. pneumoniae encodes a genetically unrelated isomerase (SP0319) that is annotated as a pentose phosphate isomerase, raising the interesting prospect that products generated by the pentose phosphate pathway may circle back and be further metabolized through the Sokatch shunt. It is likely that the modified ED pathway that occurs in enterococci, streptococci, and others, stemming from the same ancestral carnobacterial lineage, is important for generating reducing equivalents and for coping with oxidative stress from the metabolism of sugars in natural low-oxygen environments that differ from the nutrient broth used here.

In contrast to ED pathway genes, all genes for the EMP glycolysis pathway are critical or important for growth in nutrient broth (Fig. 4), as was also found for S. pneumoniae and S. aureus (17, 18), likely driven by the availability of glucose in the medium used. Similarly, all genes for the pentose phosphate pathway are critical, except for EF0197 and EF1581. In fact, in V583 EF1581 possesses a verified natural mutation rendering the transketolase (*tkt*) a pseudogene, whereas it is wild type in MMH594. That both MMH594 and V583 strains grow similarly in nutrient broth shows that taking the product past the pentose stage in this environment is not important. Given the dispensability of *tkt*, it is therefore not surprising that there is no homolog in the E. faecalis genome to the transaldolase gene (*tal*) that would interconvert the *tkt* sedoheptulose phosphate with glyceraldehyde-3-phosphate, to erythrose phosphate and fructose phosphate. This suggests that in wild-type *Enterococcus*, either sedoheptulose is a dead-end product or, perhaps more likely, as for the ED pathway, E. faecalis has an alternate way to further process the products of the ribulose phosphate epimerase reaction.

With the importance of oxidative stress management, even for growth as a standing culture in nutrient-rich medium, established by critical contributions of metalloproteins if not the ED pathway, it was of interest to determine whether other stresses pose impediments to growth in nutrient medium. Genes known or strongly suspected to be involved in all known pathways for stress management were recently compiled in a comprehensive review (11). Of the 147 genes, 59 exhibited evidence of importance for growth in Mueller-Hinton broth, including 11 identified above as contributing to metal, oxidative, and osmotic stress (Data Set S6). Moreover, 38 of those 59 lack orthologs in S. aureus and S. pneumoniae that contribute to fitness for growth in nutrient medium, highlighting different physiological challenges faced by E. faecalis. Top among those differential challenges are osmotic stresses (17 fitness-conferring genes) and acid/alkali stresses (13 fitness-conferring genes).

10.1128/mBio.02962-20.7 DATA SET S6 Tn-seq identification of stress response genes of importance for E. faecalis growth in nutrient broth. Download Data Set S6, XLSX file, 0.01 MB. Copyright © 2020 Gilmore et al. 2020 Gilmore et al. This content is distributed under the terms of the Creative Commons Attribution 4.0 International license.

### Differential aspects of the enterococcal cell envelope.

The cell envelope of enterococci differs from that of other bacteria in the composition of peptidoglycan (5), integral cell wall polymers (8, 9, 20), capsule (45), and its lipoteichoic acid (10, 46). As a result, enterococci are intrinsically resistant to lysozyme (47) and cell wall-active antibiotics (2). We therefore took four approaches for identifying genes related to the E. faecalis cell envelope. First, many important cell wall genes were previously compiled in a review on the topic (48). Second, we identified genes using an artificial intelligence-guided bioinformatic program (49). Third, we identified genes that belonged to cell wall-related COG categories and genes that were annotated as transporters. Collectively, this identified 1,199 genes as known to be or putatively related to the cell envelope. Of those, 279 exhibited evidence of importance for E. faecalis growth in Mueller-Hinton broth (Data Set S7), of which 60 were also identified in our analysis above as contributing to redox and stress response fitness. One of the largest clusters of genes shared between stress response and cell wall groups includes 17 genes associated with synthesis of the cell wall carbohydrate Epa (9, 20). These genes lie within the largest cluster (39.8 kb) of fitness-conferring genes in the E. faecalis chromosome (Data Set S3). Of the 39 genes included within this cluster, only 5 have conserved orthologs of importance in S. aureus and/or S. pneumoniae, making this the single largest block of genes uniquely important to E. faecalis.

10.1128/mBio.02962-20.8 DATA SET S7 Comprehensive identification and Tn-seq assessment of cell envelope genes of importance for E. faecalis growth in nutrient broth. Download Data Set S7, XLSX file, 0.1 MB. Copyright © 2020 Gilmore et al. 2020 Gilmore et al. This content is distributed under the terms of the Creative Commons Attribution 4.0 International license.

The structure of the Epa polymer of V583 was nicely solved in a recent report (50), which resolved long-standing questions about the relationship between Epa and E. faecalis wall teichoic acids (45). As might be expected for a large, complex carbohydrate cell wall polymer, the proposed chemistry and mechanism for synthesis have much in common with those for wall teichoic acids in other organisms, such as S. aureus. As for S. aureus, the apparent essentiality of many of the genes in the Epa operon potentially stems not from a requirement for the enzymatic product *per se* but from a blockage that leads to the lethal accumulation of biosynthetic intermediates in the membrane and resulting sequestration of various carrier molecules (51). As for the observation of the apparent essentiality of phage lysogeny controls, this warrants caution in how findings of intolerance to gene disruption on fitness are interpreted.

The largest functional categories of the 177 remaining fitness-contributing cell envelope genes that lack conserved, fitness-contributing orthologs in S. aureus and S. pneumoniae include 56 proteins of unknown function (34 being chromosomally located and 22 being associated with the pathogenicity island, phages, or other variable elements). This highlights the still-limited understanding of important differences that distinguish the rugged enterococcal cell wall from those of even closely related organisms and the more limited understanding of how that is in turn modulated by mobile elements. The largest functional category of cell envelope-associated proteins contributing to fitness are those involved in import, export, or translocation (39 proteins) (Data Set S7).

Other cell wall genes of importance to E. faecalis include eight F-type ATP synthase subunits corresponding to genes EF2607 to EF2614, which were also found to be highly essential for the fitness of S. pneumoniae in nutrient-rich medium (17). However, in S. aureus, six of the eight subunits were classified as nonessential (18). ATP synthase genes are likely nonessential in staphylococci because they possess a more fully functional electron transport chain for generating a proton gradient (52), whereas, at least *in vitro*, E. faecalis (53) and certain strains of S. pneumoniae, including TIGR4 (54), rely mainly upon the ATP synthase operon alone. Further, both S. pneumoniae and E. faecalis also harbor V-type ATP synthase machinery (genes EF1492 to EF1500), which is entirely absent in S. aureus, but neither E. faecalis nor S. pneumoniae depends on this for growth in nutrient medium in the absence of challenge.

### Genes contributing to intrinsic resistance to antibiotic challenge.

We next identified genes that contribute to intrinsic antibiotic resistance. We did this by reassessing the 2,173 MMH594 genes classified as Nonessential (dVal > 0.10) for growth in Mueller-Hinton broth for a change in their inferred importance when challenged by low-level antibiotic exposure. The E. faecalis MMH594 *mariner* insertion mutant library was exposed to 10 antibiotics at levels corresponding to 1/8 that determined to be the MIC, a level we experimentally identified as the highest level of antibiotic that does not detectably affect the growth of the population (55). Mutants were grown for approximately 12 generations, providing a dynamic range of about 5,000-fold between uninhibited growth in control cultures and a potentially total block to growth by an antibiotic. Two or three replicate cultures for each antibiotic were independently grown and compared to the 10 control replicates grown under identical conditions without antibiotics.

Genes for which control dVal was >0.100 (i.e., Nonessential) when strains were grown without challenge but <0.100 when strains were challenged with one or more antibiotics were considered candidates for being conditionally important in conferring intrinsic resistance to that antibiotic. This identified 702 of the 2,173 Nonessential genes as encoding products potentially affecting antibiotic susceptibility. However, that group was strongly enriched for genes already possessing low control dVal, suggesting that many qualify simply by already being close to the threshold used to define antibiotic effects. Therefore, as before, we used a multilayered orthogonal approach to identify a subset of candidates with the fewest possible false positives and negatives. For high-contrast comparisons (unchallenged dVal of >0.500 versus challenged dVal of <0.100), we identified genes for which a permutation test *P* value was <0.05 for comparison of the control versus challenge dVals for that gene. For medium-contrast comparisons (unchallenged dVal of >0.300), we identified genes exhibiting candidate dVals of <0.100 if the unadjusted permutation test *P* value of <0.05 was true for two or more antibiotics. For low-contrast genes (unchallenged dVal of >0.100 versus challenged dVal of <0.100), stringency was increased to minimize false-positive discovery by further adjusting the statistical *P* value threshold to <0.05 using the Benjamini-Hochberg adjustment for multiple testing. This identified 189 genes as supported by stringent statistical measures. However, previously proven or likely false negatives remained in the candidate set, including DltC (56) and CspA (57, 58). Therefore, an additional 28 genes were added to the list of resistance determinants based on an antibiotic challenge dVal of <0.100 and manual inspection of data and literature support, creating a set of 217 genes most likely to contribute to intrinsic antibiotic resistance in E. faecalis MMH594 (Data Set S8a).

10.1128/mBio.02962-20.9 DATA SET S8 (a) All genes exhibiting evidence of contribution to intrinsic antibiotic resistance; (b) intrinsic antibiotic resistance genes with greatest signal strength; (c) unique enterococcal genes showing evidence of contribution to antibiotic resistance. Download Data Set S8, XLSX file, 0.2 MB. Copyright © 2020 Gilmore et al. 2020 Gilmore et al. This content is distributed under the terms of the Creative Commons Attribution 4.0 International license.

We validated this set by testing it against all well-documented genotypes known to contribute to intrinsic resistance in E. faecalis. Penicillin-binding proteins (PBPs) have been particularly well studied (59, 60). As shown in Table 2, insertional inactivation of PBPC and PBP2B is associated with a heavy fitness penalty under all conditions. PBP2B is known to be important for septum placement and elongation of ovococci (61) and is essential for the ovococcus S. pneumoniae (62). The low-affinity penicillin-binding protein PBP4 is central to the ability of E. faecalis to tolerate cephalosporins by a known structural mechanism (63), which is in turn regulated by the two-component system CroRS/HK-RR05 and the PASTA kinase IreK (64). As predicted, inactivation of the low-affinity PBP4 results in selective sensitization to ceftriaxone in E. faecalis, which was also selectively observed for insertional inactivation of IreK. Inactivation of CroRS also exhibited the predicted cephalosporin sensitization. Interestingly, in the highly competitive Tn-seq growth environment, CroR inactivation also conferred sensitivity to other β-lactams as well as membrane-active peptides (Table 2). As also shown in Table 2, there is consistent evidence that PBP1A (EF1148) contributes to resistance to ceftriaxone and ampicillin resistance, as well as to daptomycin and trimethoprim sulfamethoxazole, and its dVal of >0.100 warranted classification in the absence of antibiotics as Nonessential. However, it is not included in the comprehensive list of genes affected by antibiotics, Data Set S8a, because the unchallenged dVal was low (0.204), providing little contrast with the conditions that reduced it below the dVal <0.100 impacted threshold, again illustrating the stringency of the inclusion criteria and the importance of considering likely statistical false negatives. In short, Tn-seq analysis using 1/8 MIC of various antibiotics showed each of the predicted known genotypes associated with cephalosporin resistance and general contributions to fitness, with notable precision. It also highlighted other potentially important contributors.

**TABLE 2:** Contribution of PBP and related genes to antibiotic susceptibility

Gene ID		Function	Fitness call	dVal (pVal) for^*a*^:										
MMH594	V583			Control	Van	Cef	Amp	Pen	Pol	Dap	T-S	Spe	Rif	Cip
SAW_00961	EF0991	PBPC	Critical	0.000	0.002 (>0.10)	0.012 (>0.10)	0.000	0.000	0.000	0.000	0.000 (>0.10)	0.052 (>0.10)	0.239 (>0.10)	0.036 (>0.10)
SAW_02652	EF2857	PBP2B	Important	0.025	0.015 (>0.10)	0.027 (>0.10)	0.057 (>0.10)	0.002 (>0.10)	0.005 (>0.10)	0.000 (>0.10)	0.000 (>0.10)	0.000 (>0.10)	0.000 (>0.10)	0.008 (>0.10)
SAW_01126	EF1148	PBP1A	Nonessential	0.204	0.513 (>0.10)	0.072 (>0.10)	0.042 (<0.10)	0.122 (>0.10)	0.131 (>0.10)	0.046 (<0.10)	0.068 (>0.10)	0.213 (>0.10)	0.158 (>0.10)	0.439 (>0.10)
SAW_02336	EF2476	PBP4	Nonessential	0.770	0.570 (>0.10)	0.028 (<0.01)	0.186 (<0.01)	0.314 (<0.05)	0.511 (>0.10)	0.148 (<0.01)	0.368 (<0.10)	0.533 (>0.10)	0.313 (<0.05)	0.288 (<0.05)
SAW_01693	EF1740	PBP1B	Nonessential	0.446	0.357 (>0.10)	1.209 (>0.10)	0.471 (>0.10)	0.419 (>0.10)	0.487 (>0.10)	0.444 (>0.10)	0.279 (>0.10)	0.549 (>0.10)	0.519 (>0.10)	0.782 (>0.10)
SAW_00671	EF0680	PBP2A	Nonessential	1.481	1.466 (>0.10)	1.168 (>0.10)	1.212 (>0.10)	1.472 (>0.10)	0.684 (<0.01)	0.304 (<0.01)	1.623 (>0.10)	1.289 (>0.10)	1.354 (>0.10)	1.506 (>0.10)
SAW_00732	EF0746	PBP(6)	Nonessential	0.437	0.692 (>0.10)	0.518 (>0.10)	0.635 (>0.10)	0.709 (>0.10)	0.466 (>0.10)	0.522 (>0.10)	0.406 (>0.10)	0.706 (>0.10)	0.635 (>0.10)	0.449 (>0.10)
SAW_02973	EF3129	PBP Lmw	Nonessential	1.701	0.986 (>0.10)	1.783 (>0.10)	1.302 (>0.10)	1.769 (>0.10)	1.689 (>0.10)	1.529 (>0.10)	1.231 (>0.10)	1.538 (>0.10)	1.336 (>0.10)	1.929 (>0.10)
SAW_02965	EF3120	IreK	Nonessential	2.218	2.570 (>0.10)	0.013 (<0.01)	2.279 (>0.10)	1.866 (<0.05)	0.179 (<0.01)	1.899 (<0.01)	2.747 (>0.10)	2.536 (>0.10)	2.107 (<0.01)	2.306 (>0.10)
SAW_00002	EF3289	CroR	Nonessential	0.166	0.143 (>0.10)	0.000 (<0.01)	0.029 (<0.05)	0.045 (<0.10)	0.000 (<0.01)	0.000 (<0.01)	0.163 (>0.10)	0.142 (>0.10)	0.046 (<0.05)	0.390 (>0.10)
SAW_00003	EF3290	CroS	Nonessential	1.035	0.765 (>0.10)	0.022 (<0.01)	0.978 (>0.10)	0.663 (<0.10)	0.00 (<0.01)	0.555 (<0.05)	1.644 (>0.10)	0.831 (>0.10)	0.223 (<0.01)	1.281 (>0.10)

a pVal, statistical likelihood of being different from control value for that gene by permutation test. Shading indicates dVal qualifying as fitness compromising (<0.100). Van, vancomycin; Cef, ceftriaxone; Amp, ampicillin; Pen, penicillin; Pol, polymyxin; Dap, daptomycin; T-S, trimethoprim-sulfamethoxazole; Spe, spectinomycin; Rif, rifampin; Cip, ciprofloxacin.

To further validate the approach, we examined the effect of *mariner* disruption of two-component signaling systems, as these systems also have been well studied. Hancock and Perego previously systematically identified (65) and then knocked out all of the two-component signaling systems in E. faecalis except HK-RR07 (EF1194/EF1193), which, in their experiments, appeared to be essential, as observed for orthologs in other systems (66). They then assessed the remaining knockouts for changes in antibiotic susceptibility and other phenotypes, finding that RR01 and RR03 insertion mutants exhibited increased sensitivity to bacitracin and that the RR05 insertion mutant showed increased susceptibility to bacitracin as well as cephalosporins. They also observed growth defects in RR04 and RR14 knockouts. Finally, it was observed by others that knocking out the equivalent of RR05 resulted in a growth defect (67). More recently, HK-RR03, also known as LiaRS, has been well studied for its contribution to daptomycin resistance in enterococci, including the involvement of a gene in a separate LiaRS-influenced operon, LiaX, in antimicrobial peptide recognition and membrane remodeling (68).

Again, Tn-seq recapitulated these results (Table 3). We found that RR07 function is essential and that HK07 gene inactivation also results in a large impairment in fitness. There is also a large fitness penalty for inactivating the HK-RR12 pair. The well-documented contribution of CroRS (HK-RR05) to cephalosporin susceptibility was mentioned above. Additionally, as found by Hancock and Perego (66), we found this system to also contribute to cyclic peptide polymyxin resistance (but not cationic peptide daptomycin resistance at the 1/8 MIC level tested). As has been well established by the Arias group, LiaRS/HK-RR03 contributes critically to the ability to tolerate daptomycin at the level tested (68). Moreover, they provided evidence that LiaX augments that, which our data may support, but the contrast between unselective and selective environments in our test was low due to a substantial fitness cost in the highly competitive Tn-seq growth environment even in the absence of antibiotic selection (Table 3). Their importance in the absence of selection indicates that, in addition to involvement in adaptation to the presence of cationic peptides reported by the Arias group (68), LiaXY likely play important roles in membrane remodeling during normal growth.

**TABLE 3:** Two-component signaling systems of importance to E. faecalis in various environments^*a*^

Gene ID		HK/R	Fitness call	dVal (pVal) for^*b*^:										
MMH594	V583			Control	Van	Cef	Amp	Pen	Pol	Dap	T-S	Spe	Rif	Cip
SAW_01169	EF1193	RR7	Critical	0.000	0.000 (NC)	0.021 (>0.10)	0.000 (NC)	0.000 (NC)	0.000 (NC)	0.000 (NC)	0.000 (NC)	0.000 (NC)	0.000 (NC)	0.000 (NC)
SAW_01170	EF1194	HK7	Important	0.019	0.000 (>0.10)	0.001 (>0.10)	0.000 (>0.10)	0.000 (>0.10)	0.000 (>0.10)	0.000 (>0.10)	0.000 (>0.10)	0.055 (>0.10)	0.000 (>0.10)	0.000 (>0.10)
SAW_00568	EF0570	HK12	Important	0.092	0.054 (>0.10)	0.072 (>0.10)	0.137 (>0.10)	0.042 (>0.10)	0.064 (>0.10)	0.150 (>0.10)	0.098 (>0.10)	0.106 (>0.10)	0.024 (>0.10)	0.032 (>0.10)
SAW_00569	EF0571	RR12	Important	0.026	0.072 (>0.10)	0.000 (>0.10)	0.000 (>0.10)	0.009 (>0.10)	0.000 (>0.10)	0.397 (>0.10)	0.004 (>0.10)	0.220 (>0.10)	0.000 (>0.10)	0.000 (>0.10)
SAW_01658	EF1703	RR4	Potentially Critical	0.007	0.002 (>0.10)	0.177 (>0.10)	0.000 (>0.10)	0.000 (>0.10)	0.000 (>0.10)	0.000 (>0.10)	0.000 (>0.10)	0.000 (>0.10)	0.000 (>0.10)	0.000 (>0.10)
SAW_01659	EF1704	HK4	Nonessential	1.929	1.533 (>0.10)	1.200 (>0.10)	0.985 (>0.10)	1.551 (>0.10)	0.934 (>0.10)	1.954 (>0.10)	1.299 (>0.10)	1.861 (>0.10)	0.805 (>0.10)	0.541 (<0.05)
SAW_00002	EF3289	RR5	Nonessential	0.166	0.143 (>0.10)	0.000 (<0.01)	0.029 (<0.05)	0.045 (<0.10)	0.000 (<0.01)	0.000 (<0.01)	0.163 (>0.10)	0.142 (>0.10)	0.046 (<0.05)	0.390 (>0.10)
SAW_00003	EF3290	HK5	Nonessential	1.035	0.765 (>0.10)	0.022 (<0.01)	0.978 (>0.10)	0.663 (<0.10)	0.000 (<0.01)	0.555 (<0.05)	1.644 (>0.10)	0.831 (>0.10)	0.223 (<0.01)	1.281 (>0.10)
SAW_01310	EF1335	HK16	Nonessential	1.164	1.224 (>0.10)	1.004 (>0.10)	0.844 (>0.10)	0.618 (>0.10)	1.286 (>0.10)	0.749 (>0.10)	0.694 (>0.10)	1.318 (>0.10)	1.391 (>0.10)	0.785 (>0.10)
SAW_01311	EF1336	RR16	Nonessential	0.139	0.005 (<0.10)	0.023 (>0.10)	0.551 (>0.10)	0.076 (>0.10)	0.297 (>0.10)	0.000 (>0.10)	0.000 (>0.10)	0.012 (>0.10)	0.000 (>0.10)	0.174 (>0.10)
SAW_02702	EF2911	RR3	Potentially Important	0.028	0.106 (>0.10)	0.194 (>0.10)	0.001 (>0.10)	0.204 (>0.10)	0.000 (>0.10)	0.000 (>0.10)	0.098 (>0.10)	0.221 (>0.10)	0.000 (>0.10)	0.448 (>0.10)
SAW_02703	EF2912	HK3	Non-essential	1.232	1.228 (>0.10)	1.568 (>0.10)	1.213 (>0.10)	1.581 (>0.10)	0.435 (<0.01)	0.000 (<0.01)	0.633 (<0.10)	0.847 (>0.10)	1.861 (>0.10)	1.237 (>0.10)
SAW_00364	EF0372	RR13	Potentially Important	0.056	0.207 (>0.10)	0.200 (>0.10)	0.023 (>0.10)	0.000 (<0.10)	0.156 (>0.10)	0.046 (>0.10)	0.078 (>0.10)	0.164 (>0.10)	0.023 (>0.10)	0.265 (>0.10)
SAW_00365	EF0373	HK13	Non-essential	1.264	1.073 (>0.10)	0.931 (>0.10)	1.096 (>0.10)	0.846 (>0.10)	1.261 (>0.10)	0.903 (>0.10)	1.246 (>0.10)	1.054 (>0.10)	1.147 (>0.10)	1.606 (>0.10)
SAW_01184	EF1209	HK14	Non-essential	1.003	0.693 (>0.10)	0.658 (>0.10)	0.693 (>0.10)	0.532 (>0.10)	0.932 (>0.10)	1.184 (>0.10)	1.348 (>0.10)	0.999 (>0.10)	0.316 (<0.05)	0.563 (>0.10)
SAW_01185	EF1210	RR14	Non-essential	0.147	0.296 (>0.10)	0.238 (>0.10)	0.199 (>0.10)	0.000 (>0.10)	0.744 (>0.10)	0.043 (>0.10)	0.097 (>0.10)	0.507 (>0.10)	0.552 (>0.10)	0.085 (>0.10)
SAW_01232	EF1260	RR6	Non-essential	1.061	1.520 (>0.10)	0.774 (>0.10)	0.243 (<0.10)	1.491 (>0.10)	0.000 (<0.01)	0.175 (<0.05)	0.544 (>0.10)	0.891 (>0.10)	0.106 (<0.05)	1.193 (>0.10)
SAW_01233	EF1261	HK6	Non-essential	1.682	1.558 (>0.10)	1.725 (>0.10)	1.648 (>0.10)	2.038 (>0.10)	0.001 (<0.01)	2.791 (>0.10)	1.323 (>0.10)	2.056 (>0.10)	0.815 (<0.10)	1.326 (>0.10)
SAW_01590	EF1632	HK17	Non-essential	3.105	2.972 (>0.10)	3.249 (>0.10)	3.962 (>0.10)	2.615 (<0.10)	3.954 (>0.10)	4.378 (>0.10)	4.008 (>0.10)	2.428 (>0.10)	3.957 (>0.10)	3.043 (>0.10)
SAW_01591	EF1633	RR17	Non-essential	0.600	0.624 (>0.10)	0.361 (>0.10)	0.134 (>0.10)	1.021 (>0.10)	0.421 (>0.10)	0.910 (>0.10)	0.825 (>0.10)	0.093 (>0.10)	0.028 (<0.10)	1.198 (>0.10)
SAW_01809	EF1863	HK8	Non-essential	0.626	0.749 (>0.10)	0.814 (>0.10)	0.756 (>0.10)	0.678 (>0.10)	0.863 (>0.10)	0.509 (>0.10)	0.720 (>0.10)	0.801 (>0.10)	0.386 (<0.10)	1.127 (>0.10)
SAW_01810	EF1864	RR8	Non-essential	0.405	0.066 (<0.10)	0.618 (>0.10)	0.751 (>0.10)	0.079 (>0.10)	0.200 (>0.10)	1.059 (>0.10)	0.250 (>0.10)	0.163 (>0.10)	0.753 (>0.10)	0.311 (>0.10)
SAW_00898	EF0926	RR9	Non-essential	2.142	0.860 (<0.05)	1.226 (>0.10)	1.583 (>0.10)	0.667 (<0.05)	0.000 (<0.01)	0.480 (<0.01)	0.433 (<0.01)	1.766 (>0.10)	1.902 (>0.10)	3.047 (>0.10)
SAW_00899	EF0927	HK9	Non-essential	2.041	1.891 (>0.10)	1.251 (>0.10)	1.977 (>0.10)	2.114 (>0.10)	0.022 (<0.01)	1.398 (>0.10)	1.164 (<0.10)	1.595 (>0.10)	0.564 (<0.01)	1.873 (>0.10)
SAW_01020	EF1050	RR10	Non-essential	4.092	4.209 (>0.10)	2.572 (<0.10)	3.700 (>0.10)	2.459 (<0.10)	2.673 (<0.10)	4.696 (>0.10)	6.319 (>0.10)	1.807 (<0.05)	2.765 (>0.10)	4.912 (>0.10)
SAW_01021	EF1051	HK10	Non-essential	4.590	5.101 (>0.10)	4.658 (<0.10)	5.733 (>0.10)	5.321 (>0.10)	5.425 (>0.10)	6.987 (>0.10)	5.602 (>0.10)	4.413 (<0.01)	4.243 (<0.01)	4.773 (<0.05)
SAW_01766	EF1820	HK15	Non-essential	0.409	0.281 (>0.10)	0.241 (>0.10)	0.650 (>0.10)	0.372 (>0.10)	0.314 (>0.10)	0.405 (>0.10)	0.711 (>0.10)	0.158 (>0.10)	0.299 (>0.10)	0.274 (>0.10)
SAW_01768	EF1822	RR15	Non-essential	1.468	0.555 (<0.10)	1.935 (>0.10)	1.227 (>0.10)	0.669 (>0.10)	1.918 (>0.10)	1.254 (>0.10)	0.816 (>0.10)	0.500 (<0.10)	0.614 (>0.10)	0.285 (<0.05)
SAW_02145	EF2218	RR1	Non-essential	1.096	0.803 (>0.10)	0.811 (>0.10)	1.091 (>0.10)	1.348 (>0.10)	0.964 (>0.10)	0.575 (<0.10)	0.718 (>0.10)	0.574 (>0.10)	0.551 (>0.10)	0.538 (<0.10)
SAW_02146	EF2219	HK1	Non-essential	14.171	15.522 (>0.10)	16.955 (>0.10)	17.565 (<0.10)	18.471 (>0.10)	16.186 (<0.05)	21.592 (>0.10)	18.225 (>0.10)	16.647 (>0.10)	17.248 (>0.10)	18.511 (>0.10)
SAW_03033	EF3196	RR2	Non-essential	0.323	0.476 (>0.10)	0.342 (>0.10)	0.784 (>0.10)	0.397 (>0.10)	0.519 (>0.10)	1.068 (>0.10)	0.698 (>0.10)	0.438 (>0.10)	0.68 (>0.10)	0.353 (>0.10)
SAW_03034	EF3197	HK2	Non-essential	1.105	1.030 (>0.10)	1.112 (>0.10)	1.036 (>0.10)	1.069 (>0.10)	1.135 (>0.10)	0.809 (>0.10)	0.718 (<0.10)	0.825 (>0.10)	0.829 (>0.10)	0.940 (>0.10)
SAW_01704	EF1752	LiaY	Important	0.021	0.000 (>0.10)	0.000 (>0.10)	0.000 (>0.10)	0.000 (>0.10)	0.000 (>0.10)	0.000 (>0.10)	0.490 (>0.10)	0.214 (>0.10)	0.065 (>0.10)	0.289 (>0.10)
SAW_01705	EF1753	LiaX	Important	0.053	0.138 (>0.10)	0.000 (>0.10)	0.096 (>0.10)	0.008 (>0.10)	0.000 (>0.10)	0.000 (>0.10)	0.000 (>0.10)	0.101 (>0.10)	0.000 (>0.10)	0.000 (>0.10)

a Light shading indicates response regulators of apparent greater importance than cognate histidine kinase; dark shading indicates dVal qualifying as fitness compromising (<0.100).

b pVal, Statistical likelihood of being different from control value for that gene by permutation test. NC, not calculated; Van, vancomycin; Cef, ceftriaxone; Amp, ampicillin; Pen, penicillin; Pol, polymyxin; Dap, daptomycin; T-S, trimethoprim-sulfamethoxazole; Spe, spectinomycin; Rif, rifampin; Cip, ciprofloxacin.

We also found evidence that, as previously reported (66), inactivation of RR04, RR05, and RR14 results in growth impairment, with that of RR04 crossing the dVal threshold for classification as a candidate for our Critical classification. RR04 represents a class of response regulators that make important contributions to fitness, whereas the sensor kinase is entirely or largely dispensable. This hybrid situation is also seen with HK-RR pairs 13, 14, 16, and 17. Reminiscent of the TA module insertion bias, in all cases, it is the response regulator that is required and the sensor kinase that is dispensable. Mechanistically, this suggests that either the default state of genes regulated by these sensor kinases is “on,” or that these response regulators can receive input signals through cross talk with other functional sensor kinases. Additional potentially important observations include the roles that HK-RR06 and HK-RR09 sensors and kinases play in defense against the cyclic peptide polymyxin and that RR17 plays in resistance to rifampin and possibly also spectinomycin (Table 3).

The well-studied Dlt operon provided a third opportunity for testing the validity of the data set. The Dlt operon encodes functions for the alanylation of cell wall components in response to environmental cues, and its activity is regulated in part by sigma factor SigV (EF3180), resulting in cell walls with reduced lysozyme susceptibility (69). Further, an inhibitor of DltA sensitized E. faecalis to cephalosporins (56). One of the targets of alanylation is lipoteichoic acid (LTA), and this modification is associated with reduced susceptibility to polymyxin and nisin (70). The Huebner group showed that deletion of one of the genes for glycosylation of diacylglycerol in LTA biosynthesis, *bgsB* (EF2890), resulted in a small increase in sensitivity to polymyxin (46). In a collaborative study (10), we recently showed that a naturally occurring loss-of-function mutation in the other glycosylating enzyme in E. faecium, BgsA (homolog of EF2891), resulted in a large increase in daptomycin susceptibility.

The Tn-seq data for the *dlt* operon and associated genes were assessed (Table 4). We recapitulated with a high degree of specificity all of the major findings related to the Dlt pathway. However, we found no contribution of SigV or OatA to fitness under any of the conditions tested, likely either because their activity relates more to peptidoglycan-related stress, such as lysozyme resistance, or because the cultures analyzed were actively dividing log-phase cells surrounded by nascent peptidoglycan yet to be modified. Transposon insertions in the *dlt* operon clearly sensitized mutants to both cephalosporin and polymyxin. Moreover, genes adjacent to the *dlt* operon, EF2751 and EF2752, showed the same polymyxin sensitization. It had been observed previously (71) that E. faecalis encoded homologs of the Bacillus subtilis bacitracin resistance uptake system BceAB. EF2752 was identified as a bacitracin permease BceB homolog that has a sensing function, and EF2049 was determined to be a structurally related permease involved in bacitracin detoxification but not sensing (71). EF2751 and EF2050 encode the ATP-binding energetic functions of these transporters. The Cook group showed that EF2751/EF2572 and EF2049/EF2050 were part of a bacitracin-inducible regulon, governed by the EF0926/EF0927 two-component system HK-RR09 (72), which is homologous to the BceAB regulator BceRS, and that all contributed to bacitracin resistance. As shown in Table 4, we recapitulated the selective involvement of HK-RR09 and the uptake-sensing EF2751/EF2752 in mediating, in this case, resistance to the alternate cyclic peptide polymyxin. However, we did not show a role for the detoxifying BceAB transporter homolog, EF2049/EF2050, most likely because we used polymyxin at 1/8 MIC, which poses no growth impediment to wild-type cells. It is entirely probable that had we tested higher levels of cyclic peptide as the Cook lab did (72), we would have generated a selective disadvantage for insertional mutations of the detoxifying pump. Both glycosylases involved in LTA biosynthesis contribute to resistance to both daptomycin and polymyxin. Furthermore, the BgsB mutation (EF2890) also appears to sensitize the cell additionally to both ceftriaxone and ampicillin. Collectively, this evidence can be organized into a coherent model based on the B. subtilis Bce system (71) and observations by the Cook group (72), as follows. EF2751/EF2752 form an uptake-sensing system for cyclic peptides, which are detected by HK-RR09. HK-RR09 then induces additional expression of EF2751/EF2752 and EF2049/EF2050 as shown by the Cook group (72), which based on evidence here then activates the *dlt* genes by a mechanism yet to be determined. Whether unmodified by Dlt d-alanylylation or by lack of glycosylation, aberrations in LTA sensitize the cell to both polymyxin and daptomycin.

**TABLE 4:** Representation of *mariner* insertions in genes known to relate to antimicrobial peptide resistance, with and without selection

Gene ID		List/aliases	Fitness call	dVal (pVal) for^*a*^:										
MMH594	V583			Control	Van	Cef	Amp	Pen	Pol	Dap	T-S	Spe	Rif	Cip
SAW_03020	EF3180	SigV	Nonessential	3.657	3.904 (>0.10)	3.875 (>0.10)	3.377 (>0.10)	3.227 (>0.10)	3.750 (>0.10)	3.831 (>0.10)	3.825 (>0.10)	3.529 (>0.10)	3.110 (>0.10)	2.254 (<0.05)
SAW_00766	EF0783	OatA	Nonessential	0.416	0.359 (>0.10)	0.29 (>0.10)	0.366 (>0.10)	0.157 (<0.10)	0.307 (>0.10)	0.306 (>0.10)	0.559 (>0.10)	0.249 (>0.10)	0.067 (<0.01)	0.375 (>0.10)
SAW_02547	EF2746	Env, Str, Genus, DltD	Nonessential	0.582	0.367 (>0.10)	0.013 (<0.01)	0.364 (>0.10)	0.399 (>0.10)	0.001 (<0.01)	0.241 (<0.10)	0.062 (<0.01)	0.324 (>0.10)	0.490 (>0.10)	0.302 (>0.10)
SAW_02548	EF2747	Env, Str, Genus, DltC	Nonessential	0.456	0.065 (>0.10)	0.000 (>0.10)	0.51 (>0.10)	0.006 (>0.10)	0.000 (>0.10)	0.107 (>0.10)	0.039 (>0.10)	0.169 (>0.10)	0.000 (>0.10)	0.370 (>0.10)
SAW_02549	EF2748	Env, Str, Genus, DltB	Nonessential	2.272	2.627 (>0.10)	0.596 (<0.01)	2.789 (>0.10)	3.807 (>0.10)	0.014 (<0.01)	1.160 (<0.01)	1.48 (<0.05)	2.837 (>0.10)	1.711 (<0.10)	3.465 (>0.10)
SAW_02550	EF2749	Env, Str, Genus, DltA	Nonessential	0.989	0.743 (>0.10)	0.032 (<0.01)	0.592 (>0.10)	0.693 (>0.10)	0.000 (<0.01)	0.085 (<0.01)	0.462 (<0.10)	0.569 (>0.10)	0.892 (>0.10)	0.577 (>0.10)
SAW_02552	EF2751	Env, BceB-sensing	Nonessential	1.432	1.232 (>0.10)	0.965 (<0.05)	1.082 (<0.10)	1.148 (>0.10)	0.000 (<0.01)	1.141 (>0.10)	1.254 (>0.10)	1.167 (>0.10)	1.353 (>0.10)	1.149 (>0.10)
SAW_02553	EF2752	Env, BceA-sensing	Nonessential	0.914	0.62 (>0.10)	0.761 (>0.10)	0.597 (>0.10)	1.399 (>0.10)	0.001 (<0.01)	1.436 (>0.10)	0.588 (>0.10)	1.343 (>0.10)	1.063 (>0.10)	0.872 (>0.10)
SAW_00898	EF0926	Str, RR09, BceR	Nonessential	2.142	0.86 (<0.05)	1.226 (>0.10)	1.583 (>0.10)	0.667 (<0.05)	0.000 (<0.01)	0.480 (<0.01)	0.433 (<0.01)	1.766 (>0.10)	1.902 (>0.10)	3.047 (>0.10)
SAW_00899	EF0927	Env, Str, HK09, BceS	Nonessential	2.041	1.891 (>0.10)	1.251 (>0.10)	1.977 (>0.10)	2.114 (>0.10)	0.001 (<0.01)	1.398 (>0.10)	1.164 (<0.10)	1.595 (>0.10)	0.564 (<0.01)	1.873 (>0.10)
SAW_01984	EF2049	BceA-detox	Nonessential	2.070	1.872 (>0.10)	2.318 (>0.10)	2.227 (>0.10)	2.038 (>0.10)	1.799 (>0.10)	1.534 (<0.05)	1.738 (>0.10)	2.471 (>0.10)	2.002 (<0.05)	1.318 (>0.10)
SAW_01985	EF2050	BceB-detox	Nonessential	4.140	4.966 (>0.10)	5.433 (>0.10)	5.295 (>0.10)	5.012 (<0.10)	5.880 (>0.10)	6.754 (>0.10)	5.74 (>0.10)	4.811 (<0.05)	6.234 (>0.10)	6.434 (>0.10)
SAW_02682	EF2890	Env, LafA, BgsB	Nonessential	0.246	0.207 (>0.10)	0.017 (<0.01)	0.054 (<0.05)	0.319 (>0.10)	0.000 (<0.01)	0.000 (<0.01)	0.135 (>0.10)	0.392 (>0.10)	0.282 (>0.10)	0.534 (>0.10)
SAW_02683	EF2891	Env, LafB, BgsA	Nonessential	0.313	0.139 (<0.10)	0.104 (<0.10)	0.164 (>0.10)	0.238 (>0.10)	0.000 (<0.01)	0.000 (<0.01)	0.424 (>0.10)	0.175 (>0.10)	0.237 (>0.10)	0.496 (>0.10)

a pVal, statistical likelihood of being different from control value for that gene by permutation test. Shading indicates dVal qualifying as fitness compromising (<0.100). Van, vancomycin; Cef, ceftriaxone; Amp, ampicillin; Pen, penicillin; Pol, polymyxin; Dap, daptomycin; T-S, trimethoprim-sulfamethoxazole; Spe, spectinomycin; Rif, rifampin; Cip, ciprofloxacin.

With validation of multiple known activities in three well-characterized systems, which established that the thresholds chosen were reasonably set and conservative, we then examined other genes among the 217 identified (Data Set S8a) for evidence of selective importance in the presence of sub-MIC antibiotics of various classes. Of the conditionally important genes that make a discernible contribution to fitness only in the presence of sub-MIC antibiotics, 105 were associated with either the cell envelope (*n* = 91), known stress response pathways (*n* = 21), and/or iron, molybdenum, and selenium uptake and metabolism (*n* = 13). This set also included 19 genes we previously found to be enriched in the pathogenic ST6 lineage compared to non-infection-associated E. faecalis strains (Data Set S8a). Recently, the Dunny group took the complementary approach of creating a well-ordered library of *mariner* mutants in most E. faecalis OG1RF genes that tolerate transposon disruption (44). To prove its utility, this collection was collectively assessed for genes that make a measurable contribution to cholic acid resistance. Of the 17 structural genes for which some evidence was found to connect to cholic acid resistance in OG1RF, only 1 overlaps with our set of MMH594 genes conditionally important in the presence of 1/8-MIC-level antibiotics, EF0370 (Data Set S8a), a gene annotated as encoding the septation ring formation regulator EzrA and here also contributing to intrinsic resistance to ceftriaxone.

Many of the genes identified as conditionally important for fitness in the presence of antibiotics are clustered within the chromosome, e.g., the *dlt* operon, providing additional insights into function and caveats to interpretation. For example, three clustered genes that become important in the presence of low-level antibiotic challenge are encoded by lysogenic phage 2, six by phage 3, three by phage 4, one by phage 5 and five by phage 6. Together with the previous observation that most phages include genes associated with lysogeny control and superinfection immunity that appear important in the absence of antibiotic stress, it is likely that this group of genes fine-tunes lysogenic control in the presences of cell stress. By analogy, this may also apply to at least some of the mobile element-conferred genes that become important on antibiotic challenge, especially those located close to the integrase/excisionase functions of those elements. This includes conditionally important genes in the pathogenicity island (PAI) (1 gene), variable island F (4 genes), variable island I (5 genes), and variable island K (2 genes). Alternatively, a few of these may augment intrinsic resistance in ways yet to be determined, which would help explain their enrichment in pathogenic lineages.

Related to known mechanisms of resistance, a cluster of genes that become important to fitness upon antibiotic exposure include a group of genes (EF1737, EF1741, EF1743, EF1745, EF1748, EF1758, and EF1769) that lie on either side of the recently described *liaXYZ* operon (EF1753–EF1751) (68). These genes have no discernible impact on daptomycin susceptibility, but, interestingly, six have dVals that suggest a contribution to cyclic peptide polymyxin resistance instead. Most also contribute to ciprofloxacin resistance as well, although the mechanistic connection is not clear. Many are hypotheticals: EF1741 encodes catabolite control protein CcpA, EF1748 encodes a prolipoprotein-modifying activity, and EF1758 and EF1769 are transporter components. When combined with the observations of the Arias group on LiaXYZ function (68) and the observation here that LiaXY are important for fitness even in the absence of selection, this suggests that this chromosomal domain encodes functions generally important for remodeling the cell membrane as carbon sources become depleted in nutrient medium or other environments, perhaps as occurs in the transition from log to stationary phase.

As noted above, the *dlt* operon also connects to cyclic peptide uptake, detection, and detoxification and is influenced by the HK-RR09/BceRS sensing system (EF0926/EF0927) located elsewhere in the chromosome. As shown in Data Set S8a, genes immediately adjacent to BceRS are also conditionally important in surviving antibiotic challenge. Interestingly, most of those genes contribute to either rifampin resistance (including EF0933, EF0938, EF0941, and EF0947) or ciprofloxacin resistance (EF0949). Additionally, EF0948 contributes to trimethoprim-sulfamethoxazole resistance. This group of genes appears to connect DNA helicity to intracellular sugar phosphate balance. With the above observations about the connection of CcpA to cell wall modification, the commonality that emerges is that EF0926/EF0927 appears to regulate expression of genes involved in metabolic transitions and, under special circumstances, in response to direct stimulation by the presence of antimicrobial peptides.

Finally, nearby is a cluster of genes related to branched-chain fatty acid biosynthesis (EF1658 to EF1690). This pathway is known to serve E. faecalis as a temporary electron sink, helping the cell achieve electron balance in the absence of the electron transport chain (73). In S. aureus, loss of branched-chain α-keto acid dehydrogenase (BKD) results in increased susceptibility to daptomycin (74). Daptomycin susceptibility was increased by mutation of five genes in this cluster (EF1665, EF1669, EF1680, EF1682, and EF1683), with two more (EF1658 and EF1666) barely missing the conservative dVal cutoff of <0.100 to be called daptomycin affected. (Another dense array of genes nearby [EF1531 and EF1592] broadly affects antibiotic susceptibility. These genes encode diverse functions, from aromatic amino acid biosynthesis to tRNA modification, and impact resistance to a variety of antibiotics, making identification of a unifying theme difficult.)

### Genes with the strongest antibiotic specific signals.

We next examined a set of 44 genes (Data Set S8b) that gave the strongest signals—genes that exhibit excellent fitness when strains in which they are mutated are grown in nutrient broth (i.e., dVal > 1.00) but profound compromise in the presence of at least one antibiotic (dVal without challenge/dVal antibiotic challenge ratio > 50). Of those, 7 are found within phages not widely shared among E. faecalis strains, leaving 37 genes of importance in this category.

When phage genes are removed, no high-signal genes remained that contributed to vancomycin resistance. For the remaining antibiotics, three genes affected ceftriaxone resistance, including those encoding CroS and IreK, discussed above, and EF0742. BLAST analysis shows that EF0742 is conserved throughout enterococci and related species (vagococci, streptococci, and lactococci) emerging from the same phylogenetic branch. A divergent homolog was studied in Lactobacillus casei and was found to share structural resemblance to SecB and to be important for surviving prolonged heat stress (75).

Polymyxin (12 genes) and daptomycin (11 genes) were the antibiotics associated with the largest numbers of high-signal-strength genes, with EF1680 contributing to resistance to both drugs. EF1680, as discussed above, occurs among genes related to branched-chain fatty acid biosynthesis. Included within this group is the adjacent gene, *msrA* (EF1681), which encodes methionine sulfoxide reductase A. MsrA and MsrB (EF3164) have been shown to be important for repairing oxidative damage and to contribute to H_2_O_2_ challenge survival (76). In the competitive growth environment of Tn-seq, insertions in either *msrA* or *msrB* are highly compromising (although it is still possible to isolate and study mutants [75]), highlighting the oxidative stress challenge enterococci experience even when growing in nutrient medium. That study further showed that EF1683 to EF1680 occur in an operon expressed in that gene order, which, along with the specificity of the effect, supports the finding that EF1680 itself is a determinant of polymyxin and daptomycin resistance (as opposed to a polar effect on EF1681).

Other genes yielding strong signals for contributing to polymyxin resistance include EF1260 (*rr06*), EF2751 (*bceB*), EF0926 (*bceR*), EF0927 (*bceS*), EF3290 (*hk05/croS*), EF2748 (*dltB*), and EF1261 (*hk06*), with known roles as discussed previously. Additionally, the K subunit of the V-type H^+^ ATPase (EF1494) contributes to polymyxin resistance, along with ampicillin and rifampin resistance. For this gene, there is a weaker signal for contributing also to vancomycin and penicillin resistance. This ATPase is encoded by a block of nine genes, EF1492 to EF1500, of which four (EF1492, EF1495, EF1496, and EF1497) and possibly more (EF1493) appear to be important for fitness even in the absence of antibiotic challenge. The importance of those genes suggests that when EF1494 is mutated, a functional complex still forms, but that complex is compromised in the presence of low levels of several antibiotics, including polymyxin. In contrast, EF1268, which is annotated as a P-type cation-transporting ATPase, E1-E2, specifically contributes only to polymyxin resistance. P-type ATPases include lipid flippases involved in membrane biogenesis (77), suggesting a possible mechanism for its contribution to polymyxin resistance.

With respect to daptomycin resistance, in addition to EF1680, nine other genes gave strong and specific signals, including that encoding LiaS. This includes EF1397, a putative molybdenum transporter that occurs in an E. faecalis-specific cluster of redox-related molybdenum metabolism genes, with several (EF1388 and EF1392 to EF1395) making important contributions to fitness even in the absence of antibiotic stress as noted previously. This again suggests that the EF1397 mutation, harmless by itself, makes the essential complex or pathway more vulnerable to daptomycin (and ampicillin and trimethoprim-sulfamethoxazole) disruption, for reasons yet to be determined. EF1889, also harmless when mutated by itself, appears to be related to the zeta toxin postsegregational killing systems of plasmids and, when mutated by insertion, sensitizes the cell to daptomycin and ampicillin.

EF1083, which sensitizes the cell to daptomycin and perhaps also to ampicillin, is of special interest, as this gene and EF1082 are ubiquitous in E. faecalis but do not occur outside this species (Data Set S1). There is no obvious source for related genes in GenBank, and their conservation throughout the species suggests an important role. Here, we found that EF1082 is likely important to fitness under all conditions. In the absence of selection, it just misses the dVal cutoff of <0.100, and it appears to be critical under exposure to all antibiotics. In contrast, EF1083 is dispensable under all conditions but is critical when exposed to low levels of daptomycin. Phyre2 did not return any high- or moderate-confidence structures for EF1082 or EF1083. These genes are adjacent to a phylogenetically more widely distributed gene, EF1084, annotated as encoding a universal stress protein. However, this gene appears dispensable for all antibiotic conditions tested, including daptomycin.

EF2149 is similarly interesting because of its ubiquity among all enterococcal species but limited occurrence outside the genus (2). Immediately adjacent, EF2150 exhibits a strong specific signal for contributing to resistance to ceftriaxone and daptomycin, but it was not included in this list because the unchallenged dVal was below the high signal threshold of dVal, >1.000 (EF2150 dVal unchallenged = 0.617); however, it is included in the broader list of antibiotic-affected genes (Data Set S8a). EF2150 encodes a tRNA-dependent lipid II-Ala + l-alanine ligase (PUBSEED) related to the FemAB family, which would explain its contribution to resistance to both classes of antibiotic. The amino-terminal half of EF2149 is predicted to form a structure similar to that of mitochondrial acyl carrier protein (Phyre2), which is involved in fatty acid biosynthesis. In contrast to widely distributed EF2150, EF2149 is conserved in all species of enterococci but does not occur outside the genus. Its specific contribution to daptomycin resistance, location adjacent to EF2150 with a similar pattern of contribution, and exclusive distribution among enterococci suggest that it modifies EF2150 activity in a manner important to a defining characteristic of all enterococci.

EF1359 sensitizes the cell to daptomycin and constitutes a DhaM-like component of phosphoenolpyruvate (PEP)-dihydroxyacetone phosphotransferase (78), which is encoded by an operon that is regulated by EF1357. In *Listeria*, an EF1357 homolog regulates 1,2 propanediol metabolism by an antisense mechanism that is in turn regulated by a riboswitch (79). Although these pathways allow growth via metabolism of glycerol and related substrates, they likely also are involved in sensing intracellular PEP levels and regulating the activities of other genes as well (80). EF0642 is another hypothetical that sensitizes the cell to daptomycin and, although ubiquitous in E. faecalis, rarely occurs outside that species.

### Genus core genes of special importance.

We previously identified a core set of 1,037 genes as shared by >90% of 24 enterococcal species (2). Of those, 126 genes distinguish enterococci from species of the genus with which it shared a most recent common ancestor, *Vagococcus*. Of those distinguishing enterococcal genes, at least 14 encode functions important for growth in nutrient broth that warranted classification here as Critical or Important. These include genes EF0394, EF0397, EF0990, EF1043, EF1146, EF2923, and EF3061 (all Fitness Critical) and EF1195, EF1316, EF1724, EF1752, EF1753, EF2606, and EF3086 (Fitness Important) (Data Set S1). Most encode unknown functions, whereas EF0394, EF0397, EF1043, EF1752, EF1753, and EF3061 appear to be extracellular or associated with the cell envelope (Data Set S7). Additionally, 16 more had no or low absolute rates of *mariner* insertion (i.e., dVal) suggestive of fitness importance, including EF1375, EF1783, EF1772, and EF2160 (Potentially Critical) and EF0954, EF1039, EF1190, EF1214, EF1250, EF1778, EF1784, EF1787, EF1790, EF1934, EF2367, and EF2664 (Potentially Impaired). Of those, EF1250, EF1375, and EF2367 appear to be extracellular or associated with the cell envelope, and EF1772, EF1778, EF1783, EF1784, EF1787, and EF1790 encode functions associated with *de novo* purine biosynthesis (Data Set S1).

Of special interest are 12 genes that, in addition to distinguishing all enterococci from *Vagococcus*, occur rarely if at all in other bacteria (Data Set S8c). Most of these genes lack known function (10/12). Half (EF0394, EF0397, EF1375, EF1190, EF1250, and EF1934) contribute to fitness in the absence of challenge and are discussed above. Genes EF1141, EF1610, EF1909, EF2149, and EF2789 contribute to fitness only in the presence of antibiotics. EF1141 is annotated as a protein in the MutT/Nudix family, enzymes that catalyze the hydrolysis of nucleoside diphosphates linked to other molecules (81). STRING connects EF1610 to the adjacent EF1611, which encodes a probable manganese-dependent inorganic pyrophosphatase. Highly divergent homologs of unknown function do occur in vagococci, lactococci, and streptococci that are below the threshold previously used to make identity calls (2), suggesting that EF1610 may have been acquired when these genera, along with the enterococci, split from carnobacterial ancestors, but the gene is under unusually heavy selection. In contrast, EF1909 is quite specific to the enterococci and essentially nothing is known about its function. Here, we show that mutation of EF1909 sensitizes the cell to all three beta-lactams tested, indicating that it contributes importantly to the cell wall structure. Of likely importance is the fact that it is immediately adjacent to EF1908, which encodes the UDP-*N*-acetylmuramate-l-alanine ligase, MurC. EF2149 shows a strong signal for daptomycin (and also ciprofloxacin) and is discussed above. EF2149 appears strictly limited to the enterococci. Finally, hypothetical protein EF2789 is rare but can be found outside enterococci, with a divergent homolog in B. subtilis, YqgQ. The structure of YqgQ has been solved and based on that structure is inferred to be a single-stranded DNA binding protein (82). Here, we found it to be specifically and strongly related to ciprofloxacin resistance, which would be consistent with that role.

In summary, validated by results consistent with virtually all that is known about intrinsic resistance of E. faecalis to cephalosporins, other beta-lactams, cyclic peptides, and daptomycin, Tn-seq provides a powerful tool for revealing relationships between genes and the intrinsic resistance of this species. In addition to supporting known relationships, the internally highly competitive nature of Tn-seq analysis provides additional insight on the relative fitness penalty associated with construction of various types of mutants, identifying those in which second-site suppressors may readily overtake a mutant population, a prospect that is rarely controlled for. In addition to identifying functions of unique importance to enterococci, we were able to shed light on a long-standing enigma about how enterococci and possibly related organisms are able to metabolize gluconate through an Entner-Doudoroff shunt without an *edd* enzyme. Finally, we were able to associate many hypothetical protein-encoding genes with phenotypes, providing insight into function and establishing a basis for prioritization in our attempt to define the distinctive nature of enterococci genetically. Although many of the identified genes have been validated by direct experimentation in the literature, despite our best efforts to take complementary approaches for extracting new insights from the data generated in a way that limits and balances the inclusion of false positives with false negatives, the role and importance of many genes identified await direct examination. This work, however, provides a rationale for prioritizing those studies.

## MATERIALS AND METHODS

### Culture conditions for Tn-seq.

To revive and ensure complete representation of the mutant pool, individual tubes of the frozen library were thawed and approximately 5 × 10^8^ CFU was used to inoculate 10 ml of brain heart infusion broth (BHI). Following revival for 4 h at 37°C, approximately 5 × 10^7^ CFU in 0.5 ml was used to inoculate 500 ml Mueller-Hinton broth, and cultures were grown for an additional 14 h to a final concentration of 5 × 10^8^ CFU/ml (∼12 generations). For challenge experiments, antibiotics were added to the final 14 h of incubation at 1/8 the MIC determined for a randomly selected pool of 10 *mariner* insertions in MMH594. This corresponded to selection with 0.125 μg/ml vancomycin, 128 μg/ml ceftriaxone, 0.25 μg/ml ampicillin, 0.5 μg/ml penicillin, 390 μg/ml polymyxin B, 0.5 μg/ml daptomycin (supplemented with 50 mg/ml CaCl_2_), 0.03125/0.15625 μg/ml trimethoprim-sulfamethoxazole, 8 μg/ml spectinomycin, 0.125 μg/ml rifampin, and 0.03125 μg/ml ciprofloxacin. 

### Library construction and sequencing.

Library preparation for Illumina sequencing was performed essentially as described previously (2, 14). Outgrowth libraries were sequenced at the Tufts University Genomics Core Facility on an Illumina HiSeq 2000 system, generating 50-bp single-end read sets. Sequencing depth for the resulting libraries corresponded to an average of 12.6 million reads per sample.

### Quality control, filtering, and correction of Tn-seq data.

Transposon junction reads were filtered and mapped by first removing poly(C) tail adapters from demultiplexed sequencing data using the FASTX toolkit (83). Reads were processed with Trimmomatic (84) to eliminate low-quality read segments. With reads mapped directly to adjacent to TA insertion sites, a relaxed leading cutoff of Phred 20 and stringent trailing cutoff of Phred 30 was used. A sliding window of size of 4 was also used to trim off read ends once an average of Phred 20 was observed. Reads with a minimum length of 15 bp were mapped to the complete E. faecalis MMH594 isolate B594 genome (GenBank assembly ID GCA_000391485.2) using bowtie2 (85) set to “very-sensitive” mode (end to end) with the maximum number of “reseed” attempts set at 10. Only the best alignments for each read, with a mapping quality of at least 10 and a maximum of 1 mismatch to the reference, were considered. Excluded from the analysis were 4,339 (1.9%) TA insertion sites, including (i) sites mapped only by true multimapping reads, or reads which align to two or more sites with equal probability, and (ii) sites with surroundings matching a nonpermissive motif recently identified to inhibit the Himar1 transposon from inserting (Fisher’s exact test *P* value of 5e−50 for a lower rate of saturation at such nonpermissive sites relative to the rest of the TA sites) (86). Read counts were normalized to correct for replication bias (16) using the BiasFactor function from the TnSeqDiff package (87) to apply a LOESS-based correction, using its default window length of 10 kb prior to downstream analysis. Of the resulting data, 3 of 35 libraries were excluded because they exhibited a correlation value below our *r* value cutoff of <0.85 compared to respective biological replicates.

### Identification of genes required for replication and growth in the absence of challenge.

Gene insertion densities and statistical values were calculated for 10 control biological replicate-derived libraries. After individual correction of the mutant counts of each sample for replication bias as described above, replicates were normalized to provide equal weight for each outgrowth using Trimmed Total Reads (TTR) (86) for scalar normalization. Because of the presence of several high-abundance outlier mutants, nonzero mutant counts were capped at the 98th percentile.

Gene insertion densities were quantified as follows. The *D* value (dVal) as determined previously (14) was modified to (i) consider only insertions within the middle 80% of genes, (ii) ignore insertions within intergenic regions transforming the primary modality of the *D* value distribution to approximately 1, and (iii) normalize expected counts per gene to the number of TA dinucleotide sites as opposed to gene length.

Multiple approaches were taken to statistically assess transposon junction read count depletion within individual genes compared to the surrounding genomic environment. A simulation-based permutation test was used to estimate the likelihood that the number of Tn insertions in a gene differed from the rate of insertion in its surrounding local genomic context by chance. We thus calculated the local insertion rate and distribution of nonzero insertion counts in regions 100 kb upstream and downstream for each gene. While replication bias correction alleviated bias in insertion counts around the chromosome, it did not address bias in saturation rates. Using a gene’s local saturation rate, the binomial distribution was used to simulate the number of saturated sites in *n* instances, where *n* was the number of TA sites observed in the middle 80% of a gene. Sites simulated as saturated were randomly assigned insertion counts from the local distribution of insertion counts observed. The total sum of insertions observed within the mid-region of each gene was compared to the sum of insertions from 100,000 simulations to calculate empirical one-sided *P* values, which were adjusted to account for multiple testing using the Benjamini-Hochberg procedure.

Of genes identified as being significantly different from their surroundings with respect to rates of *mariner* insertion, only five had dVal above 0.100 (dVal > 0.1), supporting the choice of that threshold as appropriately stringent (Data Set S1). That is, insertion rates in genes may differ from those of their surroundings by a degree that is reliably measurable by statistics (low statistical permutation test value [pVal]), but those genes nevertheless may be relatively tolerant of insertion at a rate that biologically implies minimal fitness cost to loss of that function (comparatively high dVal). Therefore, we established the dVal threshold of <0.100 as the maximum threshold for implying fitness loss and augmented that with the pVal to quantify the probability that the insertion rate in that gene differed from that of its surroundings.

Statistical assessment of transposon insertion was further refined for genes in anomalous regions of the chromosome. In order to overcome limitations in assessing statistical confidence for genes with low counts of available insertion (TA) sites, such as those occurring in clusters, especially short tRNA and rRNA genes, we adapted the above gene-centric simulation test to assess fitness impacts for groups of genes in unison. We first identified sets of contiguous genes where all members had dVal equal to or less than 0.1. Then, we modified the simulation test to assess whether a group of genes were depleted in insertions relative to their genomic context across 100,000 simulations. To avoid noise from potentially functionally irrelevant intergenic insertions, this adaptation also involved masking nongenic TA sites. Of the 185 gene groupings tested, 69 showed indications of being differentially depleted in insertions (adjusted *P* value < 0.05), and their gene constituents were thus categorized as negatively impacting cell fitness. Genes with dVal of <0.100 within these regions which were not already found to be statistically different from their surroundings were included in classifications as Fitness Critical (dVal < 0.01) or Fitness Important (0.01 < dVal < 0.1).

To capture likely false negatives from the above statistical approaches, we then considered genes with qualifying dVal (dVal < 0.010 or 0.010 < dVal < 0.100) as validated Fitness Critical or Fitness Important, respectively, if they also possessed highly conserved (E value < 10e−20) reciprocal best hit (RBH) orthologs in S. pneumoniae (17) and/or S. aureus (18) that contributed to fitness of those species under similar conditions. E. faecalis MMH594 genes products exhibiting identity to those of S. pneumoniae or S. aureus but not satisfying the RBH criteria were classified as homologs if they exhibited at least the loose E value cutoff of 1 × 10^−5^, but they were not used for promoting candidate critical or important genes.

The extent to which polar effects influenced fitness classifications was tested using a previously described approach (88). This analysis compares the number of arrangements in which fitness-critical or -impairing genes occur upstream of nonessential genes to the number downstream. After transferring operon predictions for E. faecalis V583 from DOOR2 (89) to the closely related E. faecalis MMH594 reference genome, we quantified the two types of arrangements, accounting only for adjacent genes occurring in the same operon clusters. We found roughly similar counts between the two arrangements (90 instances where fitness-critical or fitness-impairing genes were found upstream versus 78 instances downstream of nonessential genes). Thus, the data set appears to be minimally influenced by polar effects of *mariner* insertion.

Finally, we determined whether genes of particular importance to enterococci might be enriched in clustered arrangements. For this, a sliding window of 10 kb was used to scan the chromosome and calculate the proportion of TA sites within the middle 80% of genes classified as either fitness critical or fitness impairing. Windows which had at least three-quarters of the accounted TA sites falling within fitness-costly genes were reported and merged based on proximity or overlap of coordinates, identifying 16 genome regions with clustered fitness-conferring genes.

### Genes that become critical or important upon antibiotic challenge.

As for assessment of fitness for unchallenged growth, a multicriterion method combining a qualifying dVal metric with an additional statistical measure was used to identify genes contributing to intrinsic antibiotic resistance. The modified dVal for each gene was calculated for cultures challenged with 1/8 MIC of each antibiotic, based on aggregated mutant abundances across all replicates. For statistical assessment, we adapted an approach similar to the permutation test described in the TRANSIT package (86) by permuting insertion values from replicates only across samples, rather than across both insertion sites and samples. This modification allowed us to gain additional confidence for detecting differential signal arising from fewer insertion sites within genes. We were able to apply this approach because of the large number of control replicate samples (*n* = 10) in our data set. We carried out 100,000 permutations in which we shuffled the labels on treated and untreated replicates prior to computing the summed difference between treated and untreated values. The number of times a permutation resulted in a difference value which was smaller than the actual observed value for a given gene was used to increment an empirical *P* value which corresponded to the likelihood that the value for a particular gene in the challenged sample differed from the control value by chance. Compared to the original permutation test (86), this method was more sensitive at detecting conditionally essential genes while still maintaining a low accrual of false positives. We performed independent testing for each antibiotic treatment and genomic scaffold (chromosome or plasmid). Counts were normalized across all control and treated replicate data using the TTR method, as implemented in the TRANSIT package (86). Genes which harbored zero insertions across all untreated and treated samples were not tested. *P* values were adjusted for multiple testing using the Benjamini-Hochberg procedure.

Genes were then classified as contributing to antibiotic resistance if, in addition to having an unchallenged dVal of 0.100 or higher and a challenged dVal of <0.100, they met one of three additional statistical criteria: (i) for genes with a dVal of >0.5 in MH broth and a dVal of <0.1 for at least one antibiotic, the differences corresponded to an unadjusted permutation test *P* value of <0.05; (ii) for genes with a dVal of >0.3 in MH broth and a dVal of <0.1 for at least two antibiotics, the differences corresponded to an unadjusted permutation test *P* value of <0.05; and (iii) for genes with an unchallenged dVal of >0.1 in MH broth and a dVal of <0.1 for at least one antibiotic, the differences corresponded to a permutation test *P* value of <0.05 with stringent Benjamini Hochberg correction for false discovery. These statistical approaches identified 189 resistance genes. Two additional known false negatives and 26 additional genes were added to the list of conditionally important upon antibiotic selection based on manual inspection of data using criteria for repeated observations for antibiotics with related targets and/or literature support.

### Other bioinformatic and comparative genomic determinations.

The recently determined closed sequence for MMH594 isolate B594, which had not been subcultured since 1985 (12, 13), was annotated using the Broad Institute’s prokaryotic gene-calling and annotation pipeline, with transfer of gene descriptions from reference *Enterococcus* genomes enabled, as previously described (2). Temperate prophage coordinates were identified by transferring validated phage coordinates from the neighboring strain E. faecalis V583 (24). Additional coordinates for mobile genetic elements along the E. faecalis MMH594 reference genome were gathered from prior comparative genomics analyses (19, 20). TA toxin/antitoxin predictions for E. faecalis V583 were gathered from TADB 2.0 (25) using as inputs both MMH594 and the related reference genome of V583. Orthology was used to translate these annotations to the MMH594 genome. Genes involved in stress response for E. faecalis were obtained from a recent review (11).

Functional classification of genes included identification and bioinformatic prediction of proteins related to the cell envelope. For this, we compiled known and predicted chromosomal proteins related to the cell envelope of E. faecalis through both literature review (48) and bioinformatic analysis. Computational approaches included pSORTb (v3) (49), specifying the Gram-positive model, which we used to predict the subcellular localization of all chromosomal proteins and regarded all proteins categorized as localizing to the “Cell Wall” or as “Extracellular” as related to the cell envelope. Proteins categorized as localizing to the “Cytoplasmic Membrane” were also regarded as related to the cell envelope if they featured a transmembrane helix as predicted by TMHMM (90). Requiring transmembrane helices eliminated peripheral membrane proteins which might better be classified as cytoplasmic. To identify additional proteins which likely related to the cell envelope, we searched for those that either (i) were categorized as belonging to the COG functional families of cell membrane/wall/envelope biogenesis (M) or lipid transport and metabolism (I) or (ii) demonstrated substantial homology (E value < 1e−10) using BLAST (91) to a representative transporter protein from the Transporter Classification Database (92).

To assess conservation of genes among strains and species of enterococci, we searched for homologs of E. faecalis MMH594 chromosomal genes in three different genomic data sets: (i) 74 distinct sequence type representatives of E. faecalis (93); (ii) 8 diverse E. faecium strains with clinical and commensal representatives from clades A1 and B, respectively (20, 94); and (iii) 24 different species representatives from across *Enterococcus* (2). Multilocus sequence typing (MLST) analysis of E. faecalis genomes from (93) was performed using the MLST program by Torsten Seemann (https://github.com/tseemann/mlst). To search for conservation of homologous gene function with the greatest sensitivity, we used BLASTp with an E value threshold of 1 × 10^−10^. Protein-coding genes were considered core to the E. faecalis species if they were found in > 95% of the representative sequence types and core to the *Enterococcus* genus if they were found in > 80% of the representative species.

Eleven of the 74 E. faecalis sequence type representatives were regarded as clinically associated, based on their presence in previously reported clonal outbreaks (19), and three of the sequence type representatives were considered commensal because they were isolated from healthy humans (93) and their sequence types did not overlap sequence types associated with clonal outbreaks. E. faecium isolates were regarded as clinically associated if they belonged to clade A and commensal if they belonged to clade B, since population structure was shown to largely correlate to pathogenicity in the species (94).

### Data and tool availability.

Tools used for processing, mapping, and assessing the fitness cost of genes for growth on nutrient-rich medium and predicting whether genes contribute to antibiotic resistance are available at https://github.com/broadinstitute/PerMutation. Extensive and complete data sets are provided in this publication, and we are committed to providing additional raw data upon request. Short read sequencing data have been submitted to the Sequence Read Archive under BioProject PRJNA89033.
