# Supplementary figures and images for: Genes Contributing to the Unique Biology and Intrinsic Antibiotic Resistance of Enterococcus faecalis
Source: mBio. 2020 Nov 24;11(6):e02962-20. doi: 10.1128/mBio.02962-20 (PMC7701990; doi:10.1128/mBio.02962-20)

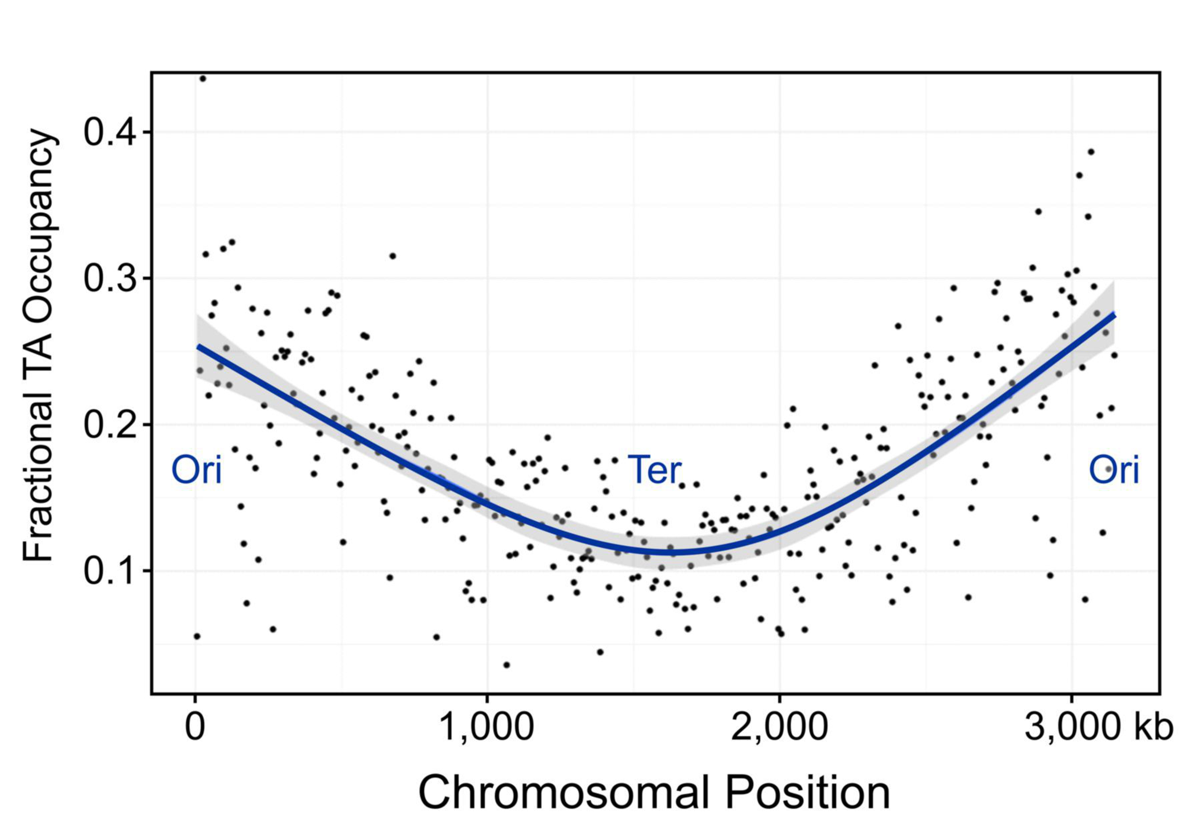

Supplement: FIG S1 [file mBio.02962-20-sf001.tif]
